# Design and Optimization of an EEG-Based Brain Machine Interface (BMI) to an Upper-Limb Exoskeleton for Stroke Survivors

**DOI:** 10.3389/fnins.2016.00122

**Published:** 2016-03-31

**Authors:** Nikunj A. Bhagat, Anusha Venkatakrishnan, Berdakh Abibullaev, Edward J. Artz, Nuray Yozbatiran, Amy A. Blank, James French, Christof Karmonik, Robert G. Grossman, Marcia K. O'Malley, Gerard E. Francisco, Jose L. Contreras-Vidal

**Affiliations:** ^1^Non-Invasive Brain Machine Interface Systems Laboratory, Department of Electrical Engineering, University of HoustonHouston, TX, USA; ^2^Mechatronics and Haptics Interfaces Laboratory, Department of Mechanical Engineering, Rice UniversityHouston, TX, USA; ^3^NeuroRecovery Research Center at TIRR Memorial Hermann and University of Texas Health Sciences CenterHouston, TX, USA; ^4^Houston Methodist Research InstituteHouston, TX, USA

**Keywords:** brain machine interface (BMI), movement related cortical potentials (MRCPs), motor intent detection, robotic exoskeleton, stroke rehabilitation

## Abstract

This study demonstrates the feasibility of detecting motor intent from brain activity of chronic stroke patients using an asynchronous electroencephalography (EEG)-based brain machine interface (BMI). Intent was inferred from movement related cortical potentials (MRCPs) measured over an optimized set of EEG electrodes. Successful intent detection triggered the motion of an upper-limb exoskeleton (MAHI Exo-II), to guide movement and to encourage active user participation by providing instantaneous sensory feedback. Several BMI design features were optimized to increase system performance in the presence of single-trial variability of MRCPs in the injured brain: (1) an adaptive time window was used for extracting features during BMI calibration; (2) training data from two consecutive days were pooled for BMI calibration to increase robustness to handle the day-to-day variations typical of EEG, and (3) BMI predictions were gated by residual electromyography (EMG) activity from the impaired arm, to reduce the number of false positives. This patient-specific BMI calibration approach can accommodate a broad spectrum of stroke patients with diverse motor capabilities. Following BMI optimization on day 3, testing of the closed-loop BMI-MAHI exoskeleton, on 4th and 5th days of the study, showed consistent BMI performance with overall mean true positive rate (TPR) = 62.7 ± 21.4% on day 4 and 67.1 ± 14.6% on day 5. The overall false positive rate (FPR) across subjects was 27.74 ± 37.46% on day 4 and 27.5 ± 35.64% on day 5; however for two subjects who had residual motor function and could benefit from the EMG-gated BMI, the mean FPR was quite low (< 10%). On average, motor intent was detected −367 ± 328 ms before movement onset during closed-loop operation. These findings provide evidence that closed-loop EEG-based BMI for stroke patients can be designed and optimized to perform well across multiple days without system recalibration.

## 1. Introduction

Functional restoration of arm and hand movements is a major goal of post-stroke rehabilitation therapy (Langhorne et al., 2009; Basteris et al., 2014). There exists evidence to suggest that robot-assisted therapy improves upper-limb functional assessment scores (Kwakkel et al., 2008; Klamroth-Marganska et al., 2014) and strength (Milot et al., 2013), by inducing activity-dependent cortical plasticity (Hogan et al., 2006; O'Malley et al., 2006; O'Dell et al., 2009). Yet, these improvements fail to reach relevant additional benefits over dose-matched conventional therapy (Kwakkel et al., 2008; Lo et al., 2010; Mehrholz et al., 2012; Klamroth-Marganska et al., 2014) or transfer into functional ability for performing daily living activities (Basteris et al., 2014). It has been suggested, that the slight benefits of robot-assisted therapy might be due to unspecific influences such as increased enthusiasm for novel interventions on the part of both patients and therapists (Kwakkel and Meskers, 2014). Notably, robotic training was less effective at restoring arm strength than conventional therapy in the study by Klamroth-Marganska et al. (2014), possibly because the device was too supportive when providing “assistance-as-needed” during the training (Chase, 2014; Brauchle et al., 2015).

Current robot-assisted therapies provide high intensity and repetitive training, but are inadequate in ensuring patient engagement, motivation, and reward, which are important factors for inducing cortical plasticity (Hogan et al., 2006; Basteris et al., 2014; Goodman et al., 2014). Therefore, recent research in robotic therapy has focused on detecting and responding to patient's motor intent, to ensure active participation of the patient during the therapy (Krebs et al., 2003; Blank et al., 2013, 2014; Hu et al., 2013). Typically, motor intent is detected via force (Kahn et al., 2006; Loureiro and Harwin, 2007; Gupta et al., 2008), or electromyography (EMG) activity (Krebs et al., 2003; Hu et al., 2009; Tong et al., 2010; Ho et al., 2011; Lenzi et al., 2012; Vaca Benitez et al., 2013) from the impaired limb's movement and the robot's motion is triggered once the intent is detected. However, these methods are only appropriate for patients who are able to produce some voluntary movement or high enough levels of muscle activity. For more severely impaired patients and to ensure patient engagement, motor intent can also be detected using noninvasive scalp electroencephalography (EEG; Wang et al., 2009; Gomez-Rodriguez et al., 2011; Frisoli et al., 2012; Venkatakrishnan et al., 2014), which is the focus of our work.

Advances in non-invasive scalp EEG have made it possible to analyze neural activity and provide feedback to the patient in real-time via a brain machine interface (BMI) through virtual and physical environments (Farina et al., 2013; Nakagome et al., 2015). Such neurofeedback can facilitate cortical plasticity and motor learning to enhance motor recovery and the resulting BMI paradigm is termed as *restorative* BMI (Soekadar et al., 2014). In this context, EEG-based restorative BMIs are easy to set up, pose no risks as compared to invasive techniques, and can be readily deployed in a clinical setting for providing rehabilitation therapy in both acute and chronic states. Hence, in recent years, several studies have proposed a neurorehabilitation regimen that augments existing robot-assisted therapy with closed-loop EEG-based BMI (Daly et al., 2008; Gomez-Rodriguez et al., 2011; Ramos-murguialday et al., 2014; Xu et al., 2014b; Ang et al., 2015) or magnetoencephalography (MEG)-based BMI (Buch et al., 2008). The BMI-Robot system usually deploys a robot or exoskeleton to command or guide the patient's movement whenever it detects the patient's voluntary motor intent. However, due to high trial-to-trial variability and poor signal-to-noise ratio (SNR) of EEG signals, detection of intent from single-trials is a daunting task (Bai et al., 2011) and poses a serious challenge to the clinical viability of EEG-based neurorehabilitation therapies. Therefore, the goal of the current study was to develop an asynchronous BMI that can detect voluntary motor intent from chronic stroke patients using EEG and command an upper-limb powered exoskeleton to provide assistance and sensory feedback. The exoskeleton used was the MAHI Exo-II (French et al., 2014), an upper-extremity exoskeleton that guided movements once intent was detected. The main focus of this feasibility study was to design and optimize an EEG-based BMI for intent detection in stroke patients, and hence we did not expect any functional changes during this short-term study.

Generally for EEG-based intent detection, either power modulations in different frequency bands (e.g., μ-rhythms, 8–12 Hz) or time domain amplitude fluctuations (e.g., slow movement related cortical potentials (MRCP) in delta band, 0.1–4 Hz) can be used. Sensorimotor (SMR) or μ-rhythms are characterized by decrease in power (desynchronization) over the contralateral sensorimotor cortex during planning and execution of imagined as well as real limb movements (Buch et al., 2008; Daly et al., 2008; Bai et al., 2011; Gomez-Rodriguez et al., 2011; Muralidharan et al., 2011b; Ramos-murguialday et al., 2014; Ang et al., 2015). In contrast, MRCPs or slow cortical potentials (SCPs) are negatively increasing potentials that occur -1.5 seconds(s) to -2 s before movement onset and reach negative peak at the onset of either self-initiated or predictably-cued movements (Cui and MacKinnon, 2009). The initial negative slope of MRCP preceding self-paced movement is often called Bereitschaftspotential (BP) or Readiness Potential (RP), whereas a similar slow negative potential observed before an imperative stimuli to externally cued movement is termed as Contingent Negative Variation (CNV) (Shibasaki and Hallett, 2006). MRCPs have been used previously to detect intention for self-paced reaching movements (Lew et al., 2012), imagined or attempted ankle dorsiflexion (Xu et al., 2014a,b), sitting and standing transitions (Bulea et al., 2014) and even for discriminating movement direction (Lew et al., 2014). BMIs that detect intent by simultaneously combining information from different types of input signals: MRCPs, μ-rhythms, and β-rhythms (Fatourechi et al., 2008; Ibáñez et al., 2014), as well as brain-neural computer interface systems which use eye movements measured via electrooculography (EOG) for interrupting unintended motion and enhance safety of an EEG-based hand exoskeleton (Witkowski et al., 2014; Soekadar et al., 2015), have also been developed.

Detecting intent from MRCPs is desirable for two reasons: (i) the magnitude and slope of MRCPs modulate with movement characteristics such as force, speed, task complexity, etc., thus providing a versatile motor control signal for capturing patient motor intent (Cui and MacKinnon, 2009; Jochumsen et al., 2013); (ii) the changes in the peak amplitude and latency of MRCPs, could potentially serve as neural indicators of cortical reorganization following motor learning and hence can further help in evaluating the efficacy of BMI-based neurorehabilitation (Yilmaz et al., 2015). Previous studies based on MRCPs have mainly dealt with healthy subjects (Bulea et al., 2014; Xu et al., 2014a,b) and/or in the case of stroke patients, have been conducted offline (Lew et al., 2012; Ibáñez et al., 2014). The brain activity of stroke patients varies to a large extent from that of a healthy intact brain, resulting in significantly differing EEG features for identical tasks (Leamy et al., 2014). Moreover, results obtained with healthy subjects rarely translate to stroke patients, and hence, it is essential to validate the closed-loop performance of MRCP-based BMI in patients with stroke. Therefore, to address this gap in the literature as well as to benefit from the aforementioned MRCP properties, we selected MRCPs for intent detection in this study.

In Section 2, our experimental procedure and methods for BMI calibration as well as for BMI control in real-time are presented. Section 3 presents the results from offline calibration and closed-loop performance using EEG-based BMI. The implications of this study are discussed in Section 4 and the conclusions are presented in Section 5. This study is registered on ClinicalTrials.gov (Identifier: NCT01948739).

## 2. Materials and methods

Four subjects (3 male) with chronic stroke participated in this study, which involved five experimental sessions (or days) per participant. The first 3 days were reserved for BMI calibration, followed by 2 days for testing closed-loop BMI control. Below we provide details for each of the components within this study. Preliminary findings in one stroke and three healthy subjects were reported in Bhagat et al. (2014).

### 2.1. Subjects

This study was carried out in accordance with the recommendations of the Institutional Review Boards of University of Houston, Rice University, University of Texas Health Science Center, and Methodist Hospital with written informed consent from all subjects. All subjects gave written informed consent in accordance with the Declaration of Helsinki. The inclusion criteria were: (1) age: 18–75 years; (2) chronic stroke (≥ 6 months post-stroke); (3) upper limb hemiparesis associated with stroke, with Manual Muscle Testing (MMT) score ranging from 2 to 4 in the elbow and wrist flexors; (4) no joint contracture or severe spasticity in the affected upper limb; (5) sufficient sitting balance to participate with robotic activities; (6) no hemineglect that would preclude participation in the study protocol; (7) no history of chemodenervation or nerve block for spasticity or pain relief to the affected limb in the past 4 months and no planned alteration in upper-extremity therapy or medication for muscle tone during the course of the study; and (8) no condition (e.g., severe arthritis, central pain) that would interfere with the administration of motor function tests. The exclusion criteria identified were: (1) orthopedic conditions of either upper extremity that would affect performance on the study; (2) untreated depression that may affect motivation to participate in the study; and (3) pregnancy.

#### 2.1.1. Post-experiment assessments

For this study, baseline clinical scores were not measured, since we did not expect them to change during the short intervention of this study. Instead, clinical and functional assessments were performed post-experiment to determine the subject's physical and cognitive impairment levels as a result of stroke. Muscle spasticity and motor impairment were evaluated using the Modified Ashworth Scale (MAS, range 0–4, 4 being maximum spasticity) and Fugl-Meyer Arm Assessment (FMA, range 0–66, 66 being normal function). MAS scores for only elbow portion of the test, i.e., flexor and extensor muscles of the affected hand are reported here, since these muscle groups were predominantly used for operating the exoskeleton. To test for cognitive impairments, the Folstein's Mini-Mental State Exam (MMSE, range 0–30, ≥ 27 implies normal cognition) was conducted. In addition, the NIH Stroke Scale (NIHSS, range 0–42, 42 meaning severe stroke impairments) was evaluated. Lastly, grip strength was measured using a hand-held dynamometer. Table 1 provides demographic details and subjects' performance on standard clinical and functional assessment tests that were conducted after completion of study. All subjects recruited were right-handed prior to onset of stroke, although S4 had used his left-hand for writing. S4 had Moyamoya disease and had suffered two strokes, ischemic followed by hemorrhagic, which occurred within a span of 1 month.

**Table 1:** **Subject demographics and clinical assessment scores**.

**Subject**	**Gender**	**Age**	**Time since**	**Stroke**	**Lesion**	**Paretic**	**MAS (Elbow)**		**FMA**	**MMSE**	**NIHSS**	**GS**
		**(years)**	**stroke (years)**	**type**	**location**	**arm**	**flexor**	**extensor**				**(%)**
S1	Male	58	5	Ischemic	Right frontal, parietal, occipital	Left	0	3	10	23	4	0
S2	Male	40	14	Hemorrhagic	Left parietal	Right	2	1+	20	29	2	6
S3	Female	68	7	Ischemic	n/a	Left	3	1	23	26	2	4
S4	Male	28	10	Ischemic + Hemorrhagic	Right frontal, parietal	Left	2	1	31	28	1	11

*MAS, Modified Ashworth Scale (range 0–4, 4 indicating maximum spasticity). Only elbow portion of the scores for flexor and extensor muscles of the affected hand are reported here*.

*FMA, Fugl-Meyer Arm score (range 0–66, higher scores representing better arm function)*.

*MMSE, Mini Mental State Exam (range 0–30, higher scores representing normal cognition)*.

*NIHSS, NIH Stroke Scale (range 0–42, higher score imply severe motor impairments)*.

*GS, Grip strength for affected hand reported as percentage of the unaffected hand score*.

*n/a, Data not available, since subject declined the MRI scan because of claustrophobia*.

T1-weighted Magnetic Resonance Images (MRI) were obtained at the Houston Methodist Research Institute MRI core using a 3T Ingenia (Philips) full body MRI scanner for the purpose of conducting source imaging. A MRI scan protocol with the following acquisition parameters was used: number of acquisitions = 1; acquisition matrix = 252 × 227; TR = 8 ms; field of view = 250 × 200; duration = 5 min, 30 s; slice thickness = 2 mm; flip angle = 8°; reconstructed in-plane resolution = 0.78 mm. Scan parameters were adjusted if necessary to account for the anatomy of the subject (such as changing the field of view or number of slices depending on the need for anatomical coverage). MRI images were acquired for all subjects, except for subject S3 who declined the MRI scan because of claustrophobia.

### 2.2. Experimental setup

#### 2.2.1. Electroencephalography (EEG)

Scalp EEG was recorded using a 64-channel, active-electrode system (actiCAP system, Brain Products GmbH, Gilching, Germany). The EEG amplifier was configured for sampling frequency = 500 Hz, resolution = 16-bit, dynamic range = ±3.2768 mV, and bandwidth = 0-1000 Hz. The EEG electrodes were positioned according to the International 10–20 system (Klem et al., 1999). The ground and reference electrodes were attached to the subject's ears, one on the unimpaired side (ground) and other on the impaired side (reference).

Four peripheral active electrodes FT9-10, TP9-10 were instead used to record EMG activity from the impaired hand. For this, the active electrodes were replaced with shielded passive electrodes using a splitter box (EIB-64A, Brain Products). Then a pair of shielded electrodes 5 cm apart (bipolar configuration) was placed on each of the biceps and triceps muscles. The EMG ground electrode was attached to the skin at the olecranon process of the unimpaired elbow joint and combined with the EEG ground at the splitter box. The amplifier range for these 4 channels was scaled to ± 327.68 mV using recording software from Brain Products. Thus, EEG and EMG signals were synchronized.

#### 2.2.2. Exoskeleton

The MAHI Exo-II has four actuated degrees of freedom (DOF), but the current study only focused on controlling a single DOF elbow joint and hence, the wrist and forearm actuators were held in a fixed position using set-point proportional-derivative control. The exoskeleton allowed adjusting the range of elbow movement for each subject within 0–60°. The exoskeleton's elbow movement was mapped to a solid green ball on the screen using a graphical user interface (GUI), for providing visual feedback. A detailed description of the exoskeleton is reported elsewhere (French et al., 2014). It was operated in two training modes for BMI calibration: user-triggered and user-driven. In the user-triggered mode, the user initiated the movement by pushing against a slight resistive force, and then the robot guided the user in performing the movement. In the user-driven mode, the user initiated and performed the movement without any guidance from the exoskeleton. Further, in the user-driven mode, the exoskeleton was back-drivable with low friction and inertia and only passively recorded the motion kinematics. As compared with the user-triggered mode, the user-driven mode required greater physical effort from the subject during the task. Consequently, subjects with excessive muscle weakness were unable to complete the task in the user-driven mode and hence for such subjects, we used the user-triggered mode only.

**Figure 1:**
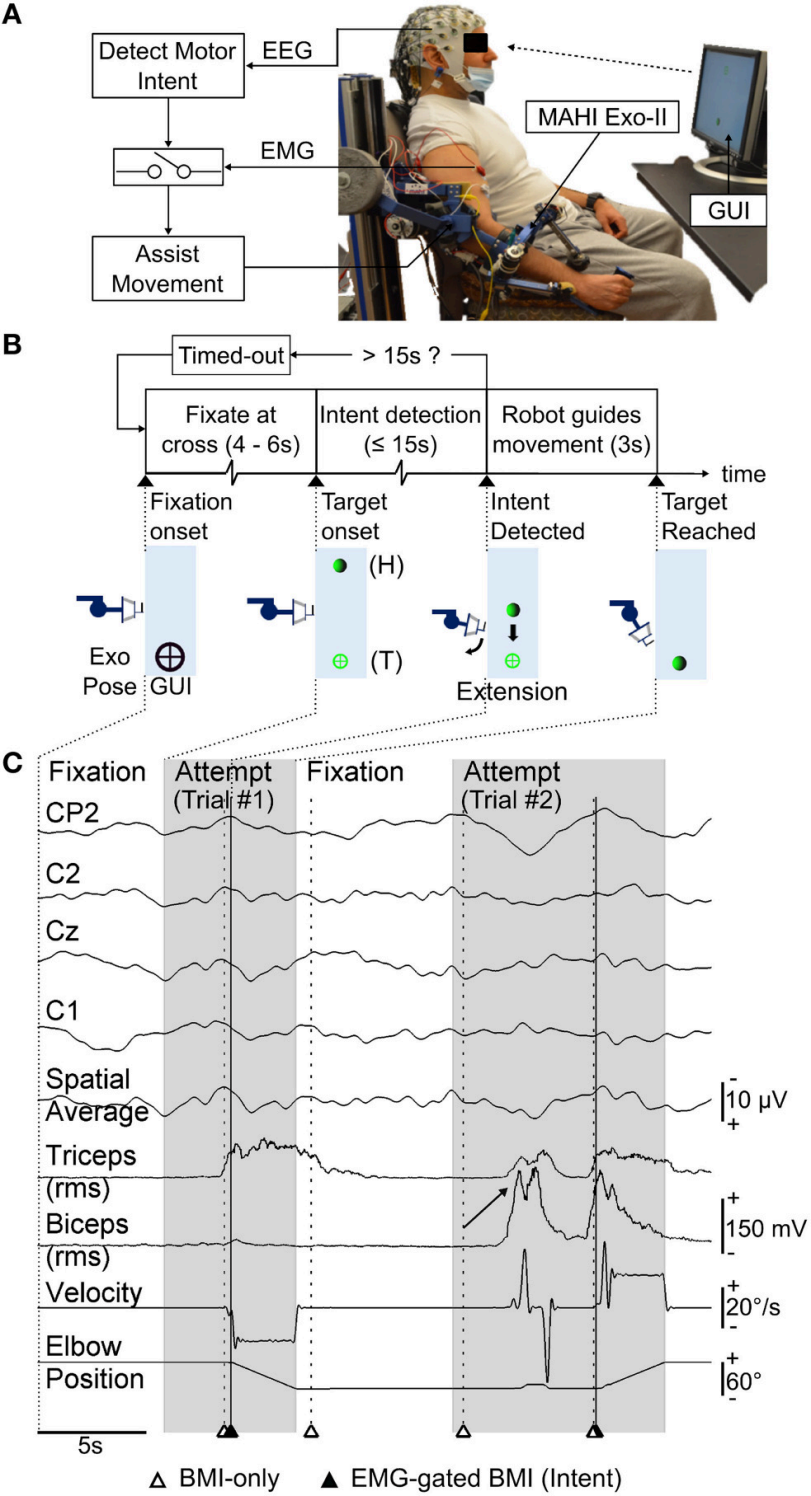
**(A)** Schematic of the asynchronous EEG-based BMI. Motor intents detected from EEG activity of a chronic upper-limb impaired stroke patient, are gated by EMG from impaired hand to trigger robot guided movement using MAHI Exo-II (currently elbow flexion/extension only). **(B)** Timeline for each trial during closed-loop BMI control task. Exoskeleton's motion (forearm extension) and corresponding visual feedback displayed on the graphical user interface (GUI) at significant events on the timeline are shown. Exoskeleton or home (H) position and target (T) position are also indicated. **(C)** Raster plot displays time-series of selected EEG (MRCP) channels and their spatial average used to detect motor intent, EMG from biceps and triceps muscles, and exoskeleton's kinematics (elbow position, velocity), during closed-loop BMI control. Alternating attempted trials (shaded) from target-onset to target-reached and fixation intervals are shown. Markers indicate BMI predictions (unfilled triangles) and successful intent detection with EMG-gated BMI (filled triangles). Trial #2 shows examples of spurious BMI-only intents i.e., false positives that were successfully rejected by EMG-gate and a missed subject attempt i.e., false negative (marked by arrow) which the BMI failed to detect. Note also the incorrect BMI-predicted motor intent during fixation interval preceding Trial #2, which was rejected by EMG-gate.

The exoskeleton's controller recorded elbow position and velocity by sampling high-resolution encoders at 1000 Hz. The exoskeleton also synchronized data capture with the EEG/EMG system by generating 5 V TTL trigger pulses. Within each trial, triggers were generated when the targets were shown (target-onset), when the subject initiated movement (movement-onset) and when a target was hit (target-reached). Movement-onset was determined during data acquisition whenever the joint velocity exceeded a predetermined threshold value. This threshold was determined on day 1 for each subject by having them move the exoskeleton for five practice trials, at a comfortable speed in user-driven mode. The threshold was then taken as 5% of the average peak velocity obtained from practice trials. For subjects that could not use the user-driven mode, the velocity threshold was heuristically adjusted until the subjects were able to comfortably initiate the exoskeleton's movement in the user-triggered mode. Figure 1A depicts the EEG-based BMI to the MAHI Exo-II exoskeleton.

#### 2.2.3. BMI calibration task

**Figure 2:**
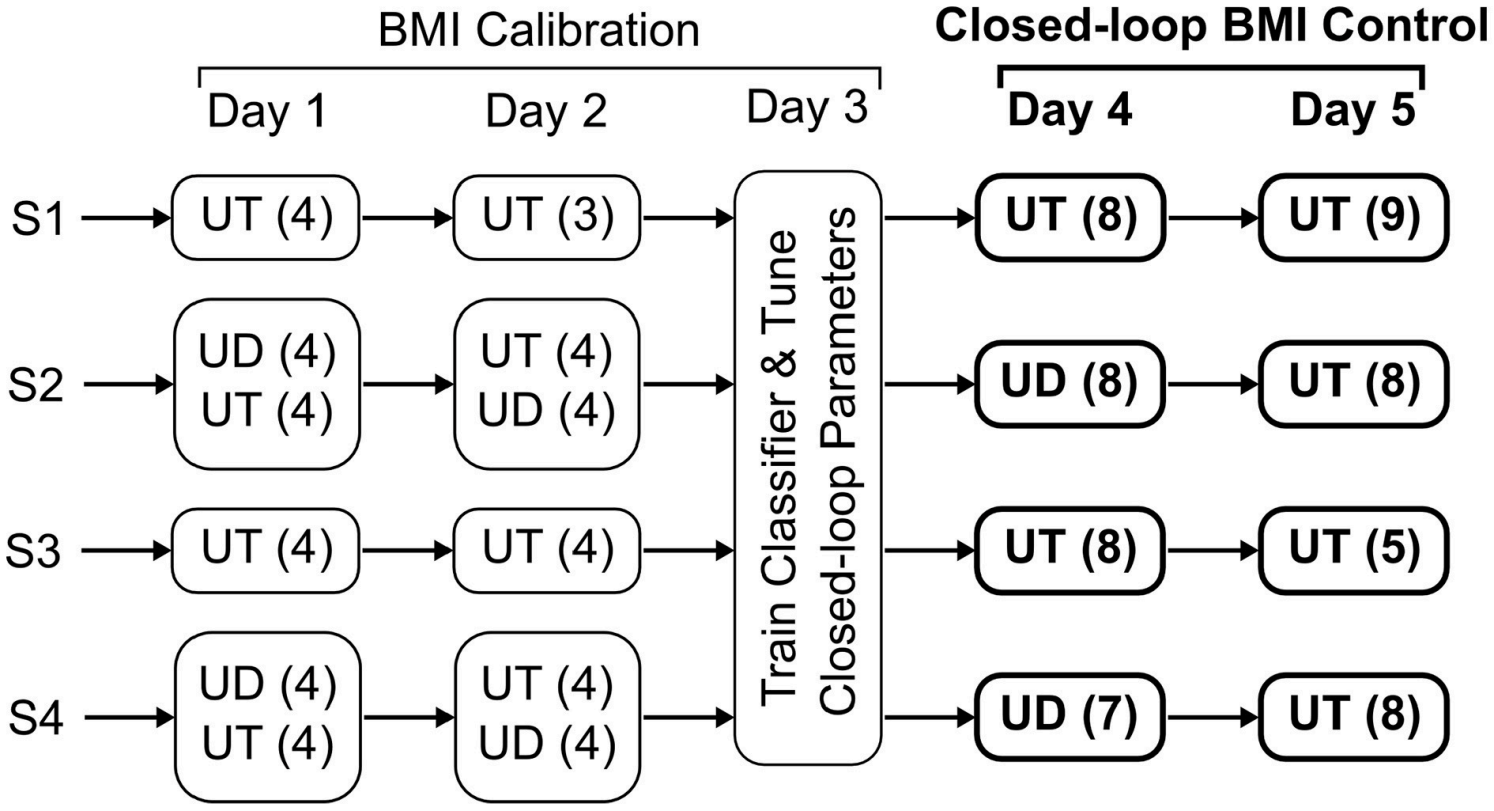
**Data collection procedure**. The parenthesis, next to the user-driven (UD) and user-triggered (UT) training modes, represent the number of blocks of 20 trials that were completed on each day.

For calibrating the BMI, subjects attempted self-initiated elbow flexion or extension to move the exoskeleton from the center position toward either an upper or lower target, respectively (Bhagat et al., 2014). The subjects were instructed to first consciously think about their preparation for the impending movement and when ready, move the exoskeleton toward the target as fast as they could. The movements were self-paced with inter-trial fixation for 4–6 s. Trials were presented in blocks of 20 and up to 8 blocks of calibration trials were recorded per day. The calibration routine was repeated on the subsequent day to account for the day-to-day EEG variability when training the BMI. Identical task design was followed for both user-driven and user-triggered modes. The data collection process was tailored depending on the subject's motor ability as shown in Figure 2. For subjects S2 and S4 that were able to use both calibration modes, we recorded 8 blocks/day (i.e., 4 consecutive blocks for each mode). On day 2, for these subjects, the order for user-driven and user-triggered modes was swapped from that of day 1. Subjects S1 and S3 could not use the user-driven mode due to excessive motor impairment and hence for them, we decided to use only the user-triggered mode for calibrating the BMI. For all subjects, on day 3, we trained a BMI classifier for each calibration mode using data from previous days and additionally fine-tuned the classifier's parameters, which were thereafter kept fixed for closed-loop BMI control.

#### 2.2.4. Closed-loop BMI control task

Once calibrated, the BMI's performance was tested in real-time during days 4 and 5. During closed-loop BMI control, the subject's goal was to use the BMI and initiate flexion or extension movement of the exoskeleton in order to reach the target. In contrast to a calibration trial, where the subjects were free to choose one of the two targets, during BMI control the target was under computer control and alternated between the two spatial positions on the screen (top or down). S1 and S3 had trained with user-triggered mode only and hence for them, we tested the same BMI classifier on both days. For S2 and S4, however, on day 4 we tested the BMI classifier that was calibrated using user-driven mode and on day 5 we tested the classifier trained using user-triggered mode. Regardless of the BMI classifier used, the BMI only triggered the movement of the exoskeleton in both modes. Hence the subjects, which were unknown to the classifier used, attempted the task in the same way.

Figures 1A,B show the closed-loop BMI implementation as well as the timeline for a typical trial during online testing. As shown in Figure 1B, the robot's current position was shown to the subjects by a solid green ball (home), whereas the fixation and target positions were shown using black and green crosses, respectively. Each trial was preceded by 4–6 s of fixation and lasted for up to 15 s during which the subjects could attempt to start exoskeleton movement using the BMI. During this span, the robot remained stationary and actively resisted any force exerted by the subject. Once the BMI detected intent, the system validated the BMI's decision by comparing it with the EMG activity from biceps and triceps of the impaired limb (Mattia et al., 2013). If EMG activity was detected in either of these muscles within 1 s following the BMI's decision, the algorithm triggered the exoskeleton to execute a pre-recorded motion sequence in order to reach the target. However, if EMG activity was absent following the BMI's decision, then the algorithm rejected the BMI's decision and did not trigger the exoskeleton's movement. The EMG-gated BMI strategy was deployed for reducing the false positives of the BMI classifier. In case the subject was unable to complete the task within the 15 s allotted, a “Timed-out” message was displayed on the screen briefly, followed by the fixation for the next trial. A raster plot of the physiological and kinematics signals along with markers for time points when the BMI had detected intent are shown in Figure 1C.

To help evaluate misclassification or false positives, a few randomly selected trials within a block were presented as “catch” trials or rare events. During a catch trial, the subjects were instructed to not think/attempt to move the robot (i.e., a planned No-go) for the entire 15 s interval. To distinguish a catch trial from a regular trial, the target was shown as a large red ball. If the EMG-gated BMI did detect intent during the catch trial it triggered the robot to move and its decision was recorded as a false positive. Each block contained from 1-5 catch trials and their order was randomized. The unbalanced ratio of catch trials (rare events) to regular trials was selected in order to allow subjects to practice BMI control of the exoskeleton and be able to learn to use the BMI for performing the movement. A balanced distribution of trials, was however maintained during offline cross-validation to get an initial estimate of classifier's performance, as described in Section 2.3.3.

On average, an entire block (i.e., 20 trials) was completed in 6.55 ± 0.64 minutes. The number of blocks completed during closed-loop BMI control varied across subjects due to subject fatigue and availability.

### 2.3. BMI decoder calibration

#### 2.3.1. Signal processing

**Figure 3:**
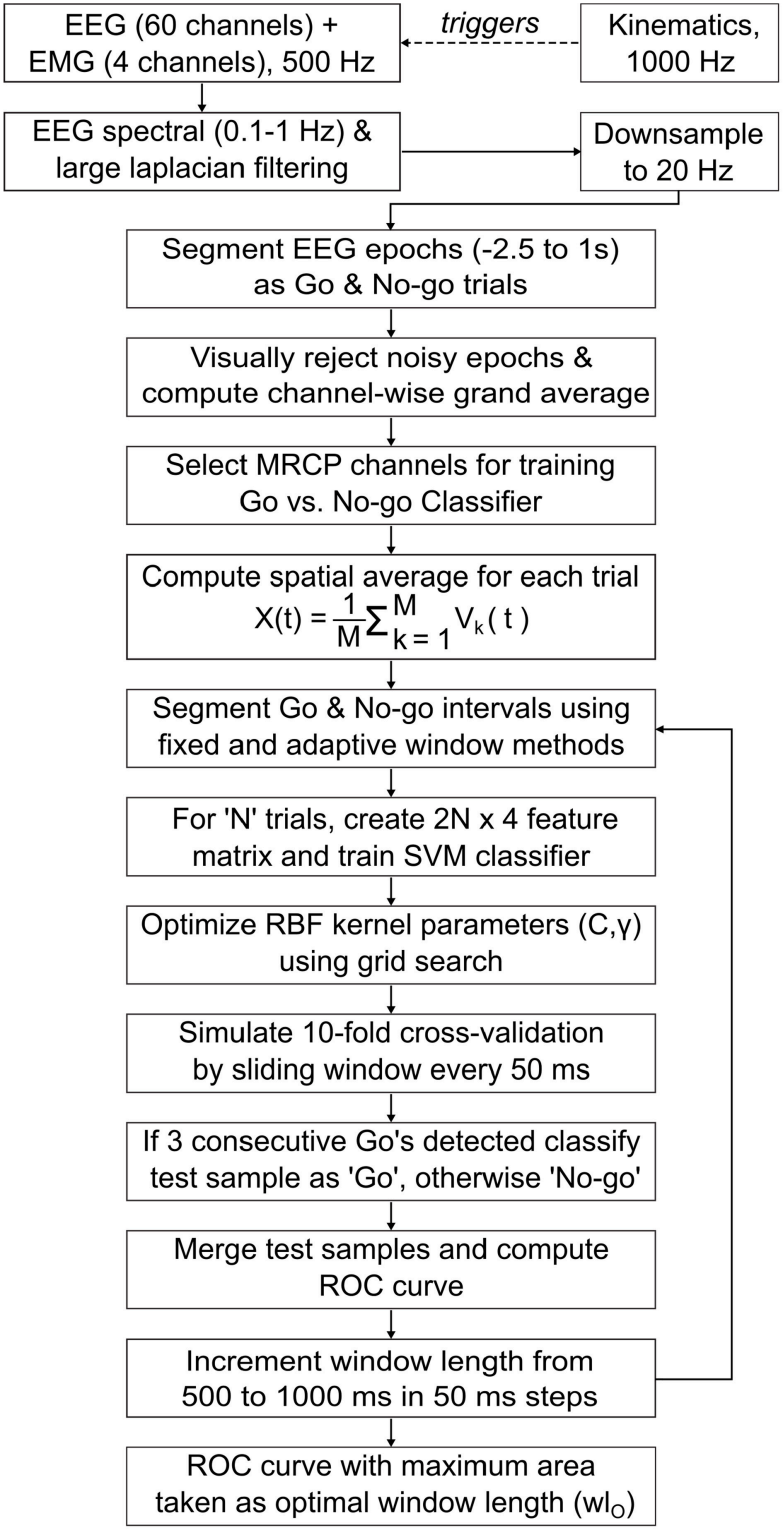
**Flowchart for offline EEG processing and classifier design**. A binary Support Vector Machine (SVM) classifier with Radial Basis Function (RBF) kernel was trained and evaluated using a simulated real-time cross-validation scheme that generated classifier prediction on test samples using a 50 ms sliding window. The classifier and optimal window length (wl_O_) that obtained in maximum area under the receiver operating characteristics (ROC) curve, were later used for closed-loop BMI implementation (see Section 2.4).

Offline data analysis was performed using MATLAB's Signal Processing and Statistics toolboxes (MATLAB, 2012), EEGLAB (Delorme and Makeig, 2004), and R Programming Language's Signal and R.matlab packages (Signal Developers, 2013; Bengtsson, 2014; R Core Team, 2014). To detect MRCPs using features extracted from EEG signals, a classifier was trained as described below and as shown in Figure 3.

EEG data recorded on days 1 and 2 were appended and filtered in the low frequency delta band (0.1–1 Hz) (Lew et al., 2012). The filters were applied in succession, i.e., initially EEG signals were high-pass filtered (causal, 4th order Butterworth, −3 dB cutoff freq. = 0.1 Hz), then re-referenced using Large Laplacian spatial filter (McFarland et al., 1997) and finally low-pass filtered (causal, 4^th^ order Butterworth, −3 db cutoff freq. = 1 Hz). Although Butterworth (IIR) filters introduce non-linear phase distortion and sometimes can be unstable, they are recommended over FIR filters when computational efficiency, sharp cutoffs and high throughput causal systems are required (Widmann et al., 2015). Also, by using a causal filter offline, we ensured that the phase distorted EEG signals used to train the classifier, would be similar to those which the classifier will encounter during real-time.

The filtered signals were downsampled to 20 Hz and segmented into epochs extending from [-2.5 s 1 s] with respect to target-onset and movement-onset triggers. Epochs aligned to movement-onset corresponded to the subject's preparation for movement, during which MRCPs are known to be generated (Shibasaki and Hallett, 2006; Cui and MacKinnon, 2009; Lew et al., 2012). Hence, these epochs (or trials) were labeled as belonging to “Go” class. Similarly, epochs aligned to target-onset were labeled as “No-go” class, since the subjects were at rest and fixating during this interval. All Go epochs were visually inspected for corruption by eye blinks or movement artifacts and the corrupted epochs were removed. For each Go epoch removed, the corresponding No-go epoch was also removed, to maintain equal class distribution. Thus, 154 ± 10 epochs per class were retained across subjects.

Next, the Go epochs were baseline corrected, by subtracting from each epoch its mean amplitude over [−2.5 s −2.25 s] interval prior to movement-onset and then time averaged, to obtain a grand-averaged waveform for each EEG channel. Baseline correction was used only during the computation of grand-averages to aid in the visualization of MRCPs and was not applied during classifier design, since it did not affect the classifier's accuracy. Previous studies show that MRCPs are observed in grand-averaged Go epochs of EEG channels over the primary motor cortex, pre-motor and supplementary motor areas (Shibasaki and Hallett, 2006; Lew et al., 2012). In addition, for stroke patients the MRCPs are distributed bilaterally over both cortices as compared to dominant contralateral distributions observed in healthy subjects (Yilmaz et al., 2015). Therefore, we visually inspected the grand-averages for channels over sensorimotor cortex and selected only those channels for which MRCPs were identified. From these channels, we manually selected a subset of channels that achieved best classification accuracy. While automated channel selection may be preferred over the manual approach taken here, a previous study found that the classifier performed equally well for both approaches (Lew et al., 2012).

Further, for each trial, the EEG epochs (*V*_*k*_(*t*)) from above selected MRCP channels (= *M*) were merged using a spatial average that is given by, $X\left(t\right)=\frac{1}{M}\overset{M}{\underset{k=1}{\sum }}V_{k}\left(t\right)$. Spatial Averaging or mean filtering is a standard image processing technique for smoothing and reducing noise in images by reducing the intensity variations in neighboring pixels (Fisher et al., 2003). We applied spatial averaging for smoothing the single-trial variations of MRCP channels and thus computed a global MRCP representation for motor intent. Trials for which the spatial averaged MRCP peaked earlier than −1.5 s before movement-onset were most likely corrupted by artifacts and such trials were removed from the training set.

#### 2.3.2. Optimal window for segmenting Go and No-go epochs

In order to extract EEG features, we segmented the Go and No-go epochs using two equal length windows. To specify a window we determined two parameters: the location of its leading edge (i.e., onset time) and its length (i.e., looking back into the past starting from onset time). Previous studies have used fixed location windows with pre-decided length. For example, in Lew et al. (2012) a fixed window from [−0.75 s −0.25 s] was used for segmenting the Go epochs, across all subjects. As shown in Section 3.2, this technique may result in poor classifier performance due to trial-to-trial variability of MRCPs. To overcome this drawback, we propose an adaptive window technique where the window location and its length for each subject is optimized to best capture the negative slope of MRCPs and compensate for its trial-to-trial variability. For this, in each trial, the location of the Go window was kept variable and made to coincide with the time when the spatially averaged MRCP reached its negative peak. Since we expected the MRCPs to be absent during fixation interval, the location of the No-go window was arbitrarily fixed at −0.5 s before target-onset.

Subsequently, the length of the Go and No-go windows were iteratively increased from 0.5 s to 1 s in steps of 50 ms. In each iteration, the classifier's performance was evaluated by computing the area under its receiver operating characteristics (ROC). Finally, the shortest window length that achieved the maximum area under the ROC curve was selected as the optimal window length (wl_O_). The window length optimization loop was also applied to the conventional fixed window technique and its performance was compared with the adaptive window technique.

#### 2.3.3. Feature extraction and classifier design

After segmenting the Go and No-go epochs, four time domain features were computed from the segmented epochs, namely slope, negative peak amplitude, area, and Mahalanobis distance. Thus, for *N* trials, we have *N* Go and No-go epochs each, resulting in a 2*N* × 4 feature matrix. The Mahalanobis distance (*d*) for each windowed epoch is calculated as its distance from the cluster of all windowed epochs belonging to the Go class. Thus,

$$d={\left[{\left(x-\mu \right)}^{⊤}\Sigma^{-1}(x-\mu )\right]}^{-\frac{1}{2}}$$

where ***x*** is a vector of signal amplitude for each Go or No-go epoch, **μ** and Σ are the mean and covariance matrix for the cluster of all Go samples (Duda et al., 2012). It is reasoned that during classification, a target or unlabeled epoch containing MRCP will be similar in shape to the known or labeled Go epochs and hence will have a smaller Mahalanobis distance (ideally 0). To minimize computation time during closed-loop BMI control, **μ** and Σ were saved during calibration and re-used later in real-time.

A binary Support Vector Machine (SVM) classifier was trained to discriminate between the Go and No-go epochs. The SVM classifier was implemented using LIBSVM library (Chang and Lin, 2011). The library's C-Support Vector Classification (C-SVC) formulation with Radial Basis Function (RBF) kernel defined as $K\left({\text{x}}_{\text{i}},{\text{x}}_{\text{j}}\right)=e^{-\gamma ||{\text{x}}_{\text{i}}-{\text{x}}_{\text{j}}|{|}^{2}},\gamma >0$ was used. The regularization and kernel parameters (C, γ) were optimized using the grid search technique for different combinations of C ϵ {10,100,1000} and γ* ϵ* {0.2, 0.5, 0.8, 1}. LIBSVM extends traditional SVM implementation and provides a probability estimate, i.e., *P*(*y* = *Go* ∣ ***x***), given a sample vector ***x*** (Chang and Lin, 2011). To classify a test sample as Go, it is required that *P*(*y* = *Go* ∣ ***x***) ≥ τ, where τ is the detection threshold (ideally τ = 0.5).

Stratified 10-fold cross-validation was used to evaluate the classifier's offline performance. During cross-validation, to test the classifier on an unseen trial, we used a sliding window that was shifted every 50 ms from [-2.5 s 1 s] with respect to either movement-onset or target-onset. The sliding window's length was set equal to the Go window length during that iteration of the optimization loop. This cross-validation scheme more closely resembled real-time BMI control by preserving the chronological order of the data and provides a more conservative estimate of accuracy than a conventional cross-validation scheme (Lew et al., 2012; Niazi et al., 2013).

As the sliding window shifted through a trial, if three consecutive windows were predicted as Go, then that trial was classified as Go. In this case, the average probability over the three consecutive Go decisions was assigned to that trial. Alternately, if the decision was No-go then the average probability over all No-go decisions within the trial, was assigned to that trial. By grouping the assigned probability estimate on all test trials from the 10 folds, the classifier's ROC curve was computed (Fawcett, 2006). The ROC curve was computed for each window length iteration and the shortest window length that resulted in the maximum area under ROC curve was chosen as optimal window length (wl_O_). After deciding wl_O_, the classifier with the highest accuracy amongst the 10 cross-validation folds for that wl_O_, was selected for closed-loop BMI implementation.

To test whether our classifier performed better than chance, we shuffled the class labels for 1000 times and for each permutation we calculated the mean classification accuracy after repeating the 10-fold cross validation. If the classifier performed better on the original training set than 95% of randomized samples, i.e., if empirical *p*−*value* < 0.05, then the difference in the mean classification accuracy was considered significant (Ojala and Garriga, 2009).

### 2.4. Closed-loop BMI implementation

For closed-loop BMI control, a custom MATLAB graphical user interface was developed that streamed EEG and EMG signals in real-time using Brain Products's streaming library. After filtering and downsampling to 20 Hz, the spatial average of selected MRCP channels was computed. A sliding window of length equal to wl_O_ generated the classifier's prediction every 50 ms. If the prediction's probability estimate exceeded the decision threshold (τ_*c*_) for *N*_*c*_ number of consecutive windows, only then the BMI made a Go decision. The parameters τ_*c*_ and *N*_*c*_ were empirically tuned on day 3 for each subject and for each calibration mode and subsequently were kept fixed on days 4 and 5.

Furthermore, to implement the EMG-gated BMI strategy, EMG signals (biceps and triceps) from the impaired hand were band-pass filtered (30–200 Hz, 8th order, Butterworth) and their root mean square (RMS) amplitude over a 300 ms interval was computed. The RMS amplitude was compared to pre-set thresholds for the biceps and triceps in order to detect EMG activity. As soon as the BMI predicted motor intent, a one second timer was started. If EMG activity was detected before the timer overflowed, then the BMI's decision was accepted and the exoskeleton performed the movement. Otherwise, the BMI's decision was rejected. EMG activity from either biceps or triceps can be used for gating both movements, i.e., initiate exoskeleton's movement, irrespective of whether the desired motion was flexion or extension.

### 2.5. Performance evaluation

True Positive Rate (TPR) and False Positive Rate (FPR) were used to evaluate the BMI's performance on days 4 and 5. TPR was defined as the fraction of attempted trials for which the motor intent was correctly detected, within each block. FPR was defined as the fraction of catch trials for which the motor intent was incorrectly detected, within each block. Two-sided Wilcoxon Rank Sum test was used to determine if the BMI's performance significantly differed between days 4 and 5. As subjects S2 and S4 attempted the task in the same way during closed-loop BMI control, regardless of whether the BMI was calibrated using user-driven or user-triggered mode, we compared the BMI's performance in their case as well.

In addition, based on the time required by the BMI to detect intent within a 15 s trial, we estimated the number of motor intents the BMI could detect per minute (min). This metric, referred simply as Intents per min = 60 × (Time(s) to detect intent)^−1^, measures the responsiveness of the BMI to the subject's motor intention. Also, we calculated the coefficient of variation (CoV) for Intents per min, to measure how dispersed their distribution was within a block. CoV was defined as the ratio of standard deviation to mean values of intents per min for a block. Furthermore, we computed the latency between motor intent detection by the BMI and the physical onset of subject's movement during closed-loop control. Physical movement onset was determined from the kinematic data, i.e., when the joint velocity exceeded a pre-set threshold.

We also asked the subjects to provide feedback on the accuracy of the BMI during closed-loop using a 5-point Likert scale. After each trial, the participants were asked: “How accurate was the BMI's decision in this trial?”. In response, the subjects provided a rated score from 1-5 where: 1-completely inaccurate, 2-moderately inaccurate, 3-not sure, 4-moderately accurate, 5-completely accurate.

Finally, to help elucidate the neural networks involved in the generation of intent in stroke patients we localized the neural signals generated in the time interval leading to the detection of motor intent during closed-loop BMI control. Cortical sources of MRCP were estimated for each subject on a trial by trial basis for days 4 and 5. The average source activation for each block and the grand-average across blocks for each day was then computed. For details on source analysis and its outcomes, refer to Supplementary Materials.

## 3. Results

### 3.1. MRCPs in stroke subjects

**Figure 4:**
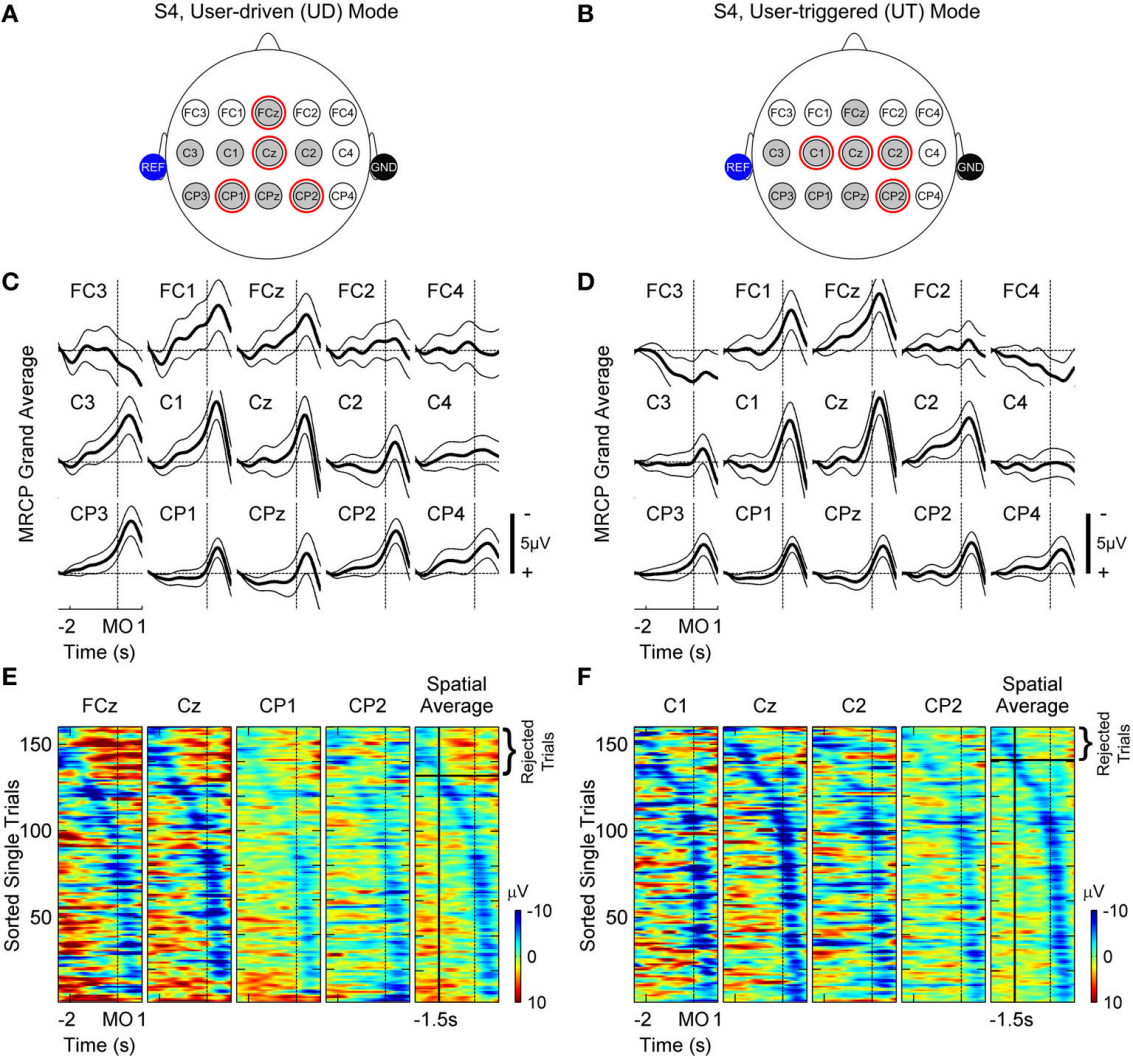
**Movement related cortical potentials (MRCPs) observed for subject S4 in user-driven (A,C,E) and user-triggered (B,D,F) modes**. **(A,B)** show a subset of EEG channels over the fronto-central, central and centro-parietal lobes, which were investigated for presence of MRCPs. Shaded gray circles represent channels for which MRCPs were observed from grand-averages in **(C,D)** and red circles highlight channels that were subsequently used for training the motor intent classifier. Shaded blue and black circles represent reference and ground electrodes respectively, which were attached to the subject's ears. **(C,D)** show baseline corrected grand-averages ± 95% confidence intervals using thick and thin black lines, respectively. In the figures, the peak of MRCP is lagging (~0.5 s) the time of movement-onset (MO) due to the non-linear phase distortion of IIR filters. **(E,F)** display raster plots of single-trial EEG amplitudes, without baseline correction, for channels used to train the classifier (columns 1–4) and their spatial average (column 5). The trials were sorted in increasing order of latency, which is defined as the time interval starting from 0.5 s up to the negative peak of spatial average. In column 5, trials for which the peak negativity of spatial average occurred earlier than −1.5 s (vertical black line) with respect to movement-onset, were rejected when training the classifier since these trials are most likely corrupted by artifacts.

Figure 4 depicts the MRCPs for subject S4. This subject was able to use both BMI calibration modes and the left and right columns correspond to the user-driven and user-triggered modes, respectively. Figures 4C,D show grand-averaged traces with 95% confidence bounds for all channels shown in Figures 4A,B. Note that the negative peak of MRCP lags by ~0.5 s with respect to movement-onset due to the non-linear phase distortion of IIR filters used for preprocessing EEG. As seen in Figures 4C,D, channels FCz, FC1, Cz, C1-C3, CPz, CP1-CP3 illustrate strong MRCPs whereas the remaining channels FC2-FC4, C4 and CP4 do not show any discernible MRCPs. Table 2 lists MRCP channels identified for all subjects. MRCP channels that were later used by the BMI classifier for detection of motor intent are marked by red circles in Figures 4A,B and shown in bold-face in Table 2. In addition, Figures 4E,F show raster plots of color-coded single-trial EEG epochs (only for Go epochs), for the selected MRCP channels and their spatial average. The raster plots were created using EEGLAB's *erpimage()* function (Delorme and Makeig, 2004). As compared to 1-D grand-averages, ERP-image plots provide a 2-D representation (epoch times × epoch amplitudes) of single-trial MRCPs and help in visualizing their inter-trial variability. The ERP-images were first sorted and then vertically smoothed using a moving average filter of length = 2 trials. The sorting order was determined from the time instant at which the spatially averaged MRCP reached a negative peak, within the interval [-2 s 0.5 s]. Epochs for which the negative peak occurred closer to 0.5 s after movement-onset, where ranked higher than other epochs, whereas epochs with negative peak occurring earlier than −1.5 s were rejected during classifier training. Table 2 shows the initial number of trials (per class) as well as the number of trials that satisfied this criterion. Amongst trials that satisfied our criteria, we found approximately equal distribution of trials between days 1 and 2. This is also indicated by the number within parenthesis in Table 2.

**Table 2:** **Optimized parameters for offline calibration and closed-loop testing of BMI control**.

**Subject**	**Calibration Mode**	**Offline calibration parameters**				**Closed-loop testing parameters**			
		MRCP channels^*^	**Initial no. of trials, per class^†^**	**No. of trials used, per class^‡^**	**wl_O_(s)**	***N*_*c*_**	**τ_*c*_**	**EMG threshold (mV)**	
								**Biceps**	**Triceps**
S1	UT	FC1, **Cz**, **C4**, **CPz**, **CP2-CP4**	134	101 (62)	0.9	2	0.425	8.5	7
S2	UD	**FCz**, **Cz**, C3, **CPz**, CP1, **CP2**, CP3, CP4	154	107 (56)	0.95	3	0.738	12	9
									
	UT	**FCz**, **FC1**, **Cz**, **C2**, CP3	160	116 (56)	0.85	5	0.72	7.5	6.4
S3	UT	**FCz**, **FC1**, **FC2**, **Cz**,**C2**,	157	105 (57)	0.9	3	0.724	44	11.5
		**C4**, CP4							
									
S4	UD	**FCz**, FC1, **Cz**, C1-C3, CPz,	160	131 (60)	0.65	6	0.735	25	25
		**CP1**, **CP2**, CP3							
	UT	FCz, FC1, **Cz**, **C1**, **C2**, C3,	160	140 (70)	0.95	5	0.723	31	21
		CPz, CP1, **CP2**, CP3							

*UD, user-driven mode; UT, user-triggered mode. Optimal window length (wl_O_) is reported here for only the adaptive window approach. τ_c_ and N_c_ are the decision thresholds for classifier's probability estimate and number of consecutive windows, respectively*.

* *Channel with bold-faced labels were later used for training the classifier*.

† *Initial no. of trials = Total calibration trials recorded − trials rejected by visual inspection*.

‡ *Number of trials eventually used for training the classifier, after rejecting trials which did not meet our criteria (see Section 2.3.1 for details). Additionally, the parenthesis indicates number of trials belonging only to day 1, which were short-listed for classifier training*.

### 3.2. Comparison of fixed and adaptive window techniques

**Figure 5:**
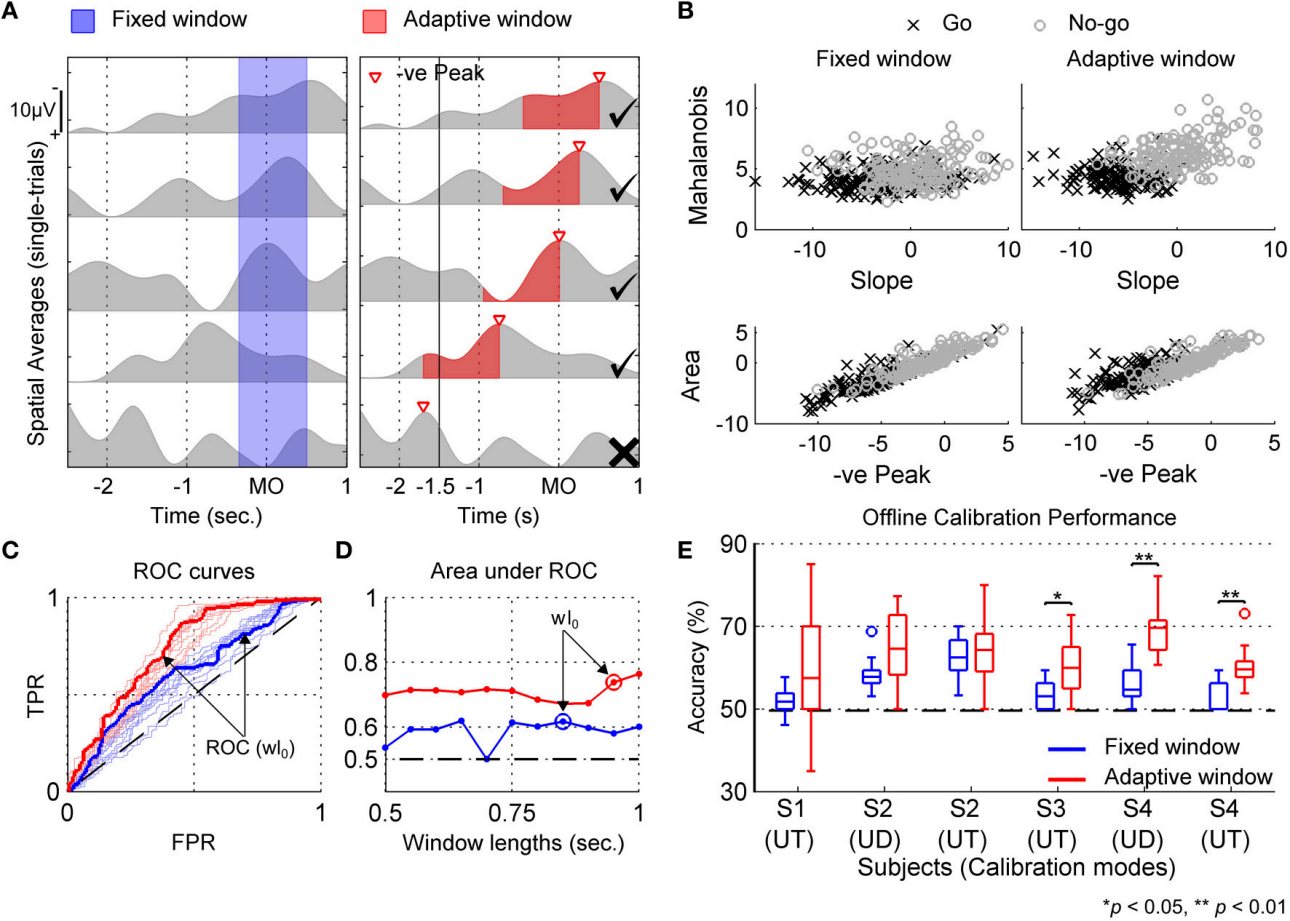
**Approach for deciding optimal window length (wl_O_) and calibrating motor intent classifier for a sample dataset (S4, user-triggered mode)**. **(A)** shows examples of single-trial spatial averaged MRCP epochs in gray superimposed with blue and red regions which represent fixed and adaptive windows defined for extracting classification features. The fixed window is predefined with respect to movement-onset (MO), whereas the adaptive window is defined for each trial with respect to negative MRCP peak. Further, for adaptive window, trials when the MRCP peaked earlier than −1.5 s were rejected from the training set (marked by **X**, otherwise by ✓). The duration of fixed and adaptive windows shown in **(A)**, correspond to wl_O_ marked in **(D)** by blue and red “o”, respectively. **(B)** Scatter plots showing the distribution of features extracted using optimal fixed and adaptive windows from Go and No-go trials. **(C)** Receiver operating characteristic (ROC) curves indicating classifier's performance in terms of True Positive Rate (TPR) and False Positive Rate (FPR) for different window lengths shown in **(D)**. Thin blue and red lines demonstrate performance curves for different fixed and adaptive window lengths, whereas bold lines indicate optimal performance curves that were obtained for each windowing technique. Random chance performance is shown by dotted black line. **(D)** shows the criteria for selecting wl_O_ based on maximum area under the ROC curve achieved. **(E)** Boxplots showing offline cross-validation accuracy for fixed and adaptive windows for all subjects and calibration modes. Statistically significant differences determined using Wilcoxon Rank Sum test are marked. The dotted black line represent the chance level accuracy, averaged across all subjects and conditions. UD, user-driven mode; UT, user-triggered mode.

Figure 5 compares the fixed and adaptive window techniques using a sample dataset (subject S4, user-triggered mode). For comparison outcomes in other subjects, refer to Supplementary Materials. In Figure 5A, a few single-trial spatial-averaged MRCP epochs from calibration data recorded for subject S4 (user-triggered mode) are shown. In the left column, a fixed window is shown that was shifted by 0.5 s after movement-onset to compensate for the filtering delays. As seen in this figure, the fixed window approach often fails to capture the negative MRCP slope in all trials and instead segments a mixture of rising and falling signal trends. Alternatively, as seen in Figure 5A (right column), the adaptive window approach consistently captures the negative slope of MRCP for each trial. Figure 5B shows the 4-D feature space using 2-D scatter plots (top and bottom), for both the fixed and adaptive windows.

Figure 5C shows the ROC curves for the classifier performance when using fixed and adaptive window techniques, whereas Figure 5D shows the optimal window length (wl_O_) selected for each technique. In addition, Figure 5E compares the 10-fold cross-validation accuracies that were obtained during calibration (offline), for each subject and calibration mode. Here accuracy refers to the percentage of correct predictions from the total predictions. Using one-sided Wilcoxon Rank Sum test, the classification accuracy for adaptive window was found to be significantly better than for fixed window for subjects S3 (*p* < 0.05) and S4 (*p* < 0.01). The median and maximum classification accuracy across all subjects was higher for adaptive window over fixed window. Higher classification accuracy is important because the classifier with the highest cross-validation fold accuracy amongst the 10 folds, was selected for closed-loop BMI implementation. Besides accuracy, the adaptive window approach also achieved larger area under the ROC curve in a majority of the cases, except for subject S2 in user-driven mode, as can be seen from Figures 5C,D and Supplementary Figures S-4–S-7. Interestingly, all classifiers were significantly better than random chance (49.6 ± 2.2%), irrespective of whether fixed or adaptive windows were used. Since the adaptive window performed better than the fixed window, we selected the classifier trained using adaptive window for closed-loop BMI control. Table 2 lists the adaptive window lengths that were optimized for each subject, as well as the closed-loop BMI parameters which were fine-tuned on day 3.

### 3.3. Closed-loop BMI performance

**Figure 6:**
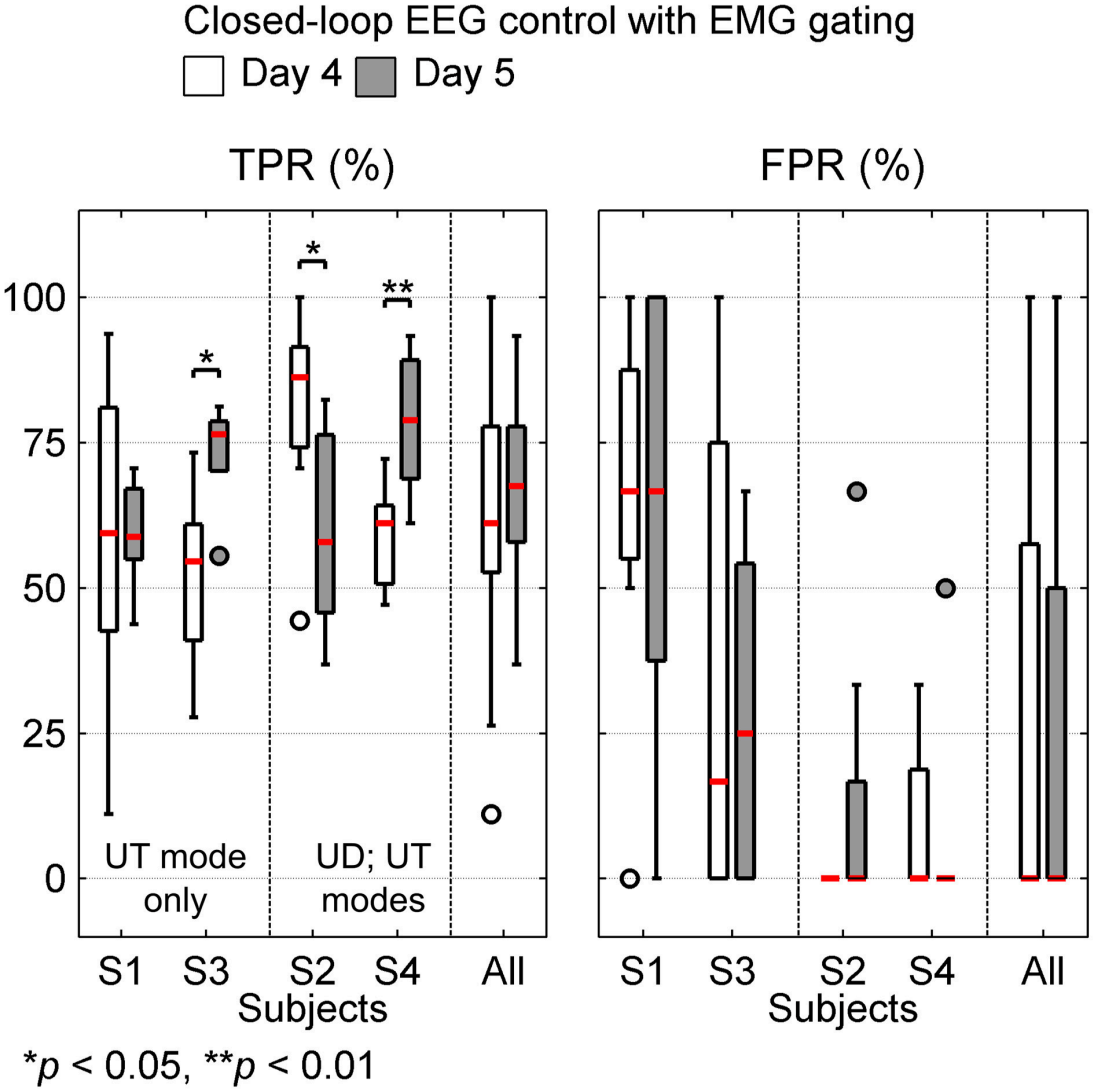
**Box plots show true positive rate (TPR) and false positive rate (FPR) for closed-loop BMI performance on days 4 and 5**. TPR and FPR were calculated on attempted trials (15–19 trials/block, overall 1063 trials) and catch trials (1–5 trials/block, overall 157 trials), respectively. The median values for BMI performance are shown in red. In each sub-plot, the last column shows the overall BMI performance achieved across all subjects. Subjects S1 and S3 using user-triggered (UT) mode on both days are grouped together. Similarly, S2 and S4 using user-driven (UD) on day 4 and user-triggered (UT) on day 5 are grouped together.

Figure 6 shows the median and interquartile range for block wise TPR and FPR that were obtained on days 4 and 5. Additionally, Table S-3 (Supplementary Materials) presents the mean ± SD values for the different metrics that were considered in this study to evaluate the closed-loop BMI performance. When considering each subject's performance individually, all subjects except S1, showed significant difference in TPR between both days. For S2, the difference was negative i.e., BMI performed very well on day 4 (user-driven mode) with maximum TPR = 100% and 0 false positives and on day 5 (user-triggered mode), however the TPR significantly decreased (*p* < 0.05) as well as the FPR marginally increased. On the other hand, for S4, the results were quite the opposite. In S4's case, the TPR on day 5 was significantly better (*p* < 0.01) than day 4 and there were only a few false positives on day 5.

**Figure 7:**
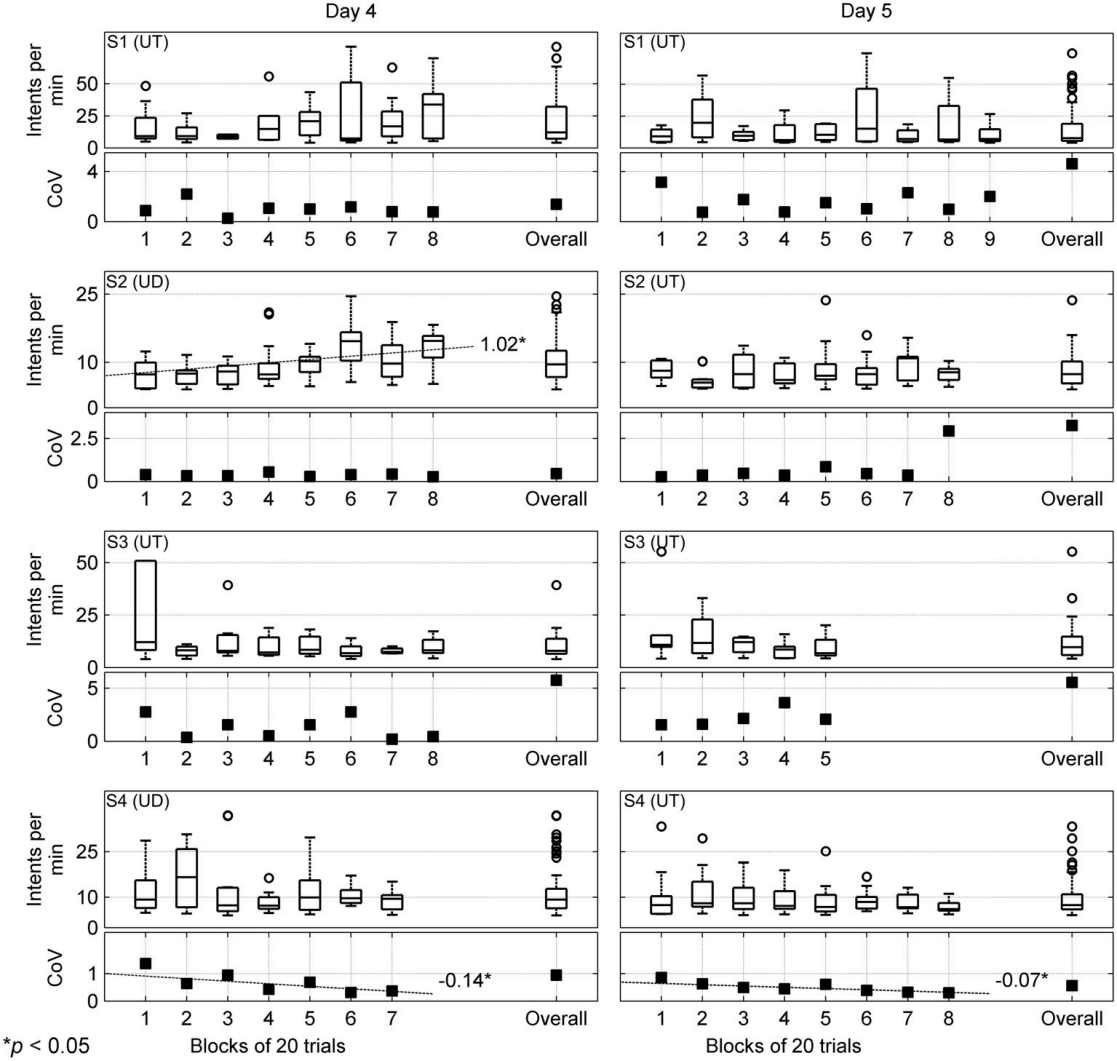
**Number of motor intents detected per min (or Intents per min) and its coefficient of variation (CoV) are computed for each block of 20 trials that tested the closed-loop BMI control and are shown here for days 4 and 5 by the left and right columns, respectively**. The subject names and calibration modes are shown in the top left corner for each row. Each row consists of a box plot displaying number of intents per min and a plot showing their CoV for each block. Additionally, within each plot the overall intents per min and CoV that were computed by combining performance of all blocks for that day is shown. Outliers are represented by “°”; however few outliers outside the axes range are not shown. The dotted lines show statistically significant trends in the median values for intents per min and individual values of CoV across all blocks for that day, along with their slopes. UD, user-driven mode; UT, user-triggered mode.

To further understand how the BMI's performance evolved within each session and across both sessions, we estimated the number of intents per minute. In Figure 7, the block wise intents per min for days 4 and 5 are shown. Within each block, the intents per min were calculated for only those trials for which the BMI correctly detected intent. Underneath each boxplot for intents per min, we plot the block wise coefficient of variation (CoV). The overall distribution of intents per min and its CoV for each day is shown by accumulating the values obtained for that day.

In general, the number of intents detected per min by the BMI largely fluctuated across blocks. However, for S2 (day 4), using regression analysis we found that the median values for intents per min significantly increased across blocks. Since, within a session the BMI was kept fixed, this suggests that within a single session, with repeated practice S2 had learned to effectively control the BMI. Moreover, the increase in intents per min also corroborates well with our previous result when we found that for S2 on day 4, the BMI performed almost perfectly. A similar trend was also found for S1 on day 4, which tended towards significance (*p* = 0.055). Overall on both days, the median number of intents detected per min hovered around 7-12. The CoV estimates remained fairly uniform for S1 (day 4) and S2 (days 4 and 5). However, for S4 we found that on each day, the CoV significantly decreased as the subject practiced with more blocks. This suggests that with more block repetitions, the variance in BMI's performance decreased and it was able to consistently detect the subject's intent. The subjects' rating of the BMI performance averaged 3.15 ± 1.68 over the two days of closed-loop testing, indicating the subjects felt the BMI system responded on average to their intent (Table S-3, Supplementary Materials).

## 4. Discussion

In this study we designed and optimized an asynchronous EEG-based BMI to perform goal-oriented movements using an upper extremity powered exoskeleton (MAHI Exo-II). The feasibility of the BMI system was validated in four chronic stroke patients over two days. The proposed BMI can be calibrated using either the user-driven or user-triggered modes of the exoskeleton, to accommodate patients with varying levels of motor impairment. Further, the BMI's false positive rate was substantially reduced by incorporating an EMG-gate as a ground truth for the subject's motor intentions. The BMI paradigm was designed to be asynchronous such that the subjects were free to attempt the trial any time after the target appeared (in fact, an instruction stimulus informed the subjects that they could start the volitional trial any time they wished) and before the trial timed out, while the BMI was continuously analyzing the ongoing brain activity (Leeb et al., 2007). This approach differs from a synchronous BMI, wherein the EEG is analyzed in predefined time intervals and the participants are instructed to imagine their movement following a auditory cue presented by the system (Brauchle et al., 2015). While both asynchronous and synchronous are feasible for the current application, the former approach provides more flexibility by allowing the user to control the timing of the exoskeleton's movement or otherwise remain idle (Leeb et al., 2007).

The overall performance across all subjects, combined over both days was TPR = 64.86 ± 18.35% and FPR = 27.62 ± 36.37%. Also, the mean TPR on day 5 (67.08 ± 14.55%) was consistent with the mean TPR for day 4 (62.71 ± 21.43%). Although the mean FPR including all subjects was 27.74 ± 37.46 on day 4 and 27.5 ± 35.64 on day 5, when considered individually, S2 and S4 had very low FPR (< 10%). As seen from Table 1, subjects S1 and S3 are older and more severely impaired (mean age = 63 years, mean FMA score = 16.5), as compared to S2 and S4 (mean age = 34 years, mean FMA score = 25.5). Moreover, due to excessive motor impairment, S1 and S3 were unable to use the user-driven mode and their EMG signals were weak and unreliable. These factors could have contributed to excessive FPR in these subjects. However, for S1, while there was no change in the median TPR and FPR on both days, the variability in TPR reduced considerably on day 5. This was also true for S3, where in fact the TPR on day 5 significantly (*p* < 0.05) improved over day 4. This suggests that these subjects were adapting well to the BMI paradigm, despite their severe motor impairment and possibly age-related cognitive decline.

In Table 3, we compare our results with previous offline and online BMI studies that have tested EEG-based intent detection, specifically with stroke patients. While a majority of the online BMI studies have focused on sensorimotor rhythms (SMR) for detecting intent, we observed comparable performance using MRCPs. Interestingly, using MRCPs alone we were able to achieve offline true positive rates (82 ± 16%) matching that of an hybrid (SMR + MRCP) BMI (82 ± 10%) which was proposed in Ibáñez et al. (2014).

**Table 3:** **Review of EEG-based motor intent detection studies in stroke patients**.

	**References**	**Studies**	**No. stroke subjects**	**Method**	**Accuracy mean (SD)%**
**Offline**	Muralidharan et al., 2011b	Muralidharan et al., 2011	4	SMR	TPR_*max*_ = 70 FPR = 22 (9)
	Antelis et al., 2012	Antelis et al., 2012	4	SMR	71 (10)
	Lew et al., 2012	Lew et al., 2012	2^a^	MRCP	TPR_*max*_ = 79 (12) FPR = 10
	Niazi et al., 2013	Niazi et al., 2013	5^a^	MRCP	TPR = 60 (11) FPR/min = 4 (4)
	Ibáñez et al., 2014	Ibáñez et al., 2014	6^a^	SMR + MRCP	TPR = 82 (10) FPR/min = 1.5 (1)
	–	Current study [offline]	4	MRCP	TPR^c^ = 82 (16) FPR^c^ = 44 (18)
**Online**	Buch et al., 2008	Buch et al., 2008	8	SMR, [MEG]	73 (18), [median]
	Daly et al., 2008	Daly et al., 2008	3	SMR	82-98
	Ang et al., 2011	Ang et al., 2011	11	SMR	82 (−)^b^
	Gomez-Rodriguez et al., 2011	Rodriguez et al., 2011	2^a^	SMR	84 [AUC]
	–	Current study [online]	4	MRCP	TPR = 65 (18) FPR = 28 (36)

*SMR, Sensorimotor or μ-rhythms; MRCP, Movement related cortical potentials; AUC, performance reported as area under the ROC curve*.

a *Study included both healthy and stroke participants. Here we mention only the number of stroke participants. If available, we report only the BMI accuracy that was obtained with stroke patients*.

b *Study reported both online and offline accuracies. Here we only consider online accuracy*.

c *Overall TPR and FPR computed offline during BMI Calibration. Note that EMG gating was not used when computing offline accuracy*.

In addition to online intent detection accuracy, the latency for intent detection is also a significant factor in determining the clinical viability of BMI-based neurorehabilitation therapy. Ideally, the intent for movement should be detected well in advance to allow a casual and seamless transfer from motor intention to movement execution via the exoskeleton (Grosse-Wentrup et al., 2011; Niazi et al., 2013). Moreover, the concomitant activation of the motor cortex during movement planning and the afferent sensory feedback provided by the exoskeleton is necessary for inducing neural plasticity as per Hebbian theory (Grosse-Wentrup et al., 2011; Muralidharan et al., 2011a).

Therefore, it is encouraging that the proposed BMI was able to detect intent before actual movement onset in nearly all subjects (Table S-3, Supplementary Materials). The overall detection latency across both days was −367 ± 328 ms prior to the subjects' physical movement onset. These results are comparable to the latencies reported in previous studies with stroke and healthy subjects: −620 ± 250 ms (Bai et al., 2011), −460 ± 85 ms (Lew et al., 2012), −152 ± 238 ms (Niazi et al., 2013), −317 ± 73 ms (Jochumsen et al., 2013), etc. and support the feasibility of detecting motor intent in patients with stroke using MRCPs.

The EMG-gated BMI approach, presented in this study, acts like a logical AND between the BMI and EMG predictions and hence its performance represents a lower bound on the TPR and FPR of the EMG-only condition, i.e., the TPR/FPR for EMG-only condition will be at least as much as EMG-gated BMI or higher. To confirm this, for subjects S2 and S4 who had residual motor function and could benefit from EMG-gating, an offline analysis was performed to compute the TPR and FPR when considering an EMG-only controller. The results were for S2, on day 4, TPR = 91 ± 10%; FPR = 4 ± 11% and on day 5, TPR = 88 ± 9%; FPR = 33 ± 44%. For S4, on day 4, TPR = 80 ± 9%; FPR = 17 ± 19% and on day 5, TPR = 89 ± 6%; FPR = 6 ± 17%. The higher FPR obtained in the EMG-only condition for S2 (days 4 and 5) and S4 (day 4) occurs because it uses simple thresholding as compared to the conservative classification approach applied by EMG-gated BMI (see Table S-3, Supplementary Materials). The trade-off however, is that the TPR of the EMG-gated BMI is also reduced. Thus, as compared to using an EMG-only controller, the EMG-gated BMI approach improves the specificity of intention detection at the cost of reduced sensitivity.

It is interesting to note that some evidence of operant conditioning of neural activity may have occurred in S2 wherein a linear increase in number of motor intents was detected across sessions (Figure 7). The combination of visual and proprioceptive feedback associated with robot-assisted arm movement could have promoted increased volitional control of movement-related cortical activity in the patient. While seen only in one patient in our initial feasibility study, this finding is particularly interesting as it provides additional support to the hypothesis that BMI-assisted robotic rehabilitation therapies can trigger neural plasticity, similar to the findings of (Naros and Gharabaghi, 2015). In our future clinical trial, we plan to further study and leverage the effect of such operant conditioning to enhance effectiveness of each training session.

Previous studies have found that MRCPs occur bilaterally over the scalp during motor preparation, and gradually become more lateralized before and during the movement execution (Platz et al., 2000; Shibasaki and Hallett, 2006). Thus, in our application, bilateral activation for detection of motor intent was expected. Furthermore, due to the maladaptive higher involvement of the unaffected hemisphere during motor preparation of paretic hand, ipsilateral over-activation (i.e., higher negative amplitudes) and a contralateral lower activation can be observed (Yilmaz et al., 2015). This was also observed in the current study in Figures 4A,B for subject S4, who was impaired in the left-hand. Therefore, we used brain activity over both hemispheres for implementing the BMI. This approach differs from conventional restorative BMIs that rely completely on ipsilesional brain activity (Soekadar et al., 2014). However, the afferent sensory feedback provided by exoskeleton movement is provided to the affected arm, thus encouraging patients to actively participate in the therapy and thereby achieve better functional recovery (Daly and Wolpaw, 2008; Venkatakrishnan et al., 2014).

### 4.1. Trial-to-trial variability of MRCPs

In the literature, different signal processing and machine learning techniques have been proposed to improve SNR of EEG signals and reduce variability of MRCPs for single-trial intent detection. In Garipelli et al. (2013), the authors propose an optimal spectral filter with pass-band [0.1–1 Hz] and a combination of common average reference and smoothening spatial filters for preprocessing EEG signals, followed by Linear Discriminant Analysis (LDA). Alternately, a high dimensional time-embedded feature matrix, which at each time point incorporates MRCP samples from up to 50 ms in the past, followed by dimensionality reduction and classification using Gaussian Mixture Models, has been proposed in Bulea et al. (2014). Yet another approach, combines high dimensional spatio-temporal ERP features and subsequently classifies into target vs. non-target using either regularized-LDA or multiple Logistic Regressors (Blankertz et al., 2011; Marathe et al., 2014). Interestingly, in all the above studies for extracting features for training the classifier, a fixed window was used. In this study, we addressed this issue by proposing an adaptive window technique for extracting MRCP features during classifier training.

Single-trial EEG variability has been traditionally attributed to changes in background neural activity and other non-neural artifacts (Blankertz et al., 2011; Garipelli et al., 2013). However, it is possible that the temporal and amplitude variability in EEG reflects changes in task performance, neural adaptation/learning and endogenous changes in global brain state due to fluctuations in sustained attention, fatigue, etc. (Goldman et al., 2009; Marathe et al., 2014). Studies examining the relation between MRCPs and movement speeds have found that for faster movements, the onset of MRCP (or BP) was delayed and it peaked sooner than for slower movements (Shibasaki and Hallett, 2006; Gu et al., 2009). These findings suggest that single-trial variability of MRCPs could also be influenced by the subject's volition to select the movement speed and direction for a trial. In addition, changes in fatigue and attention can introduce variability in EEG, especially if the same task is repeated over several trials. Although a detailed analysis of this conjecture is outside the scope of this paper, it led to the design of the adaptive window technique, for minimizing the effect of MRCP variability on the classifier's performance.

### 4.2. Study limitations

One potential limitation of the current study is the effect of artifacts on the classifier's performance. In this study however, we use low frequency, narrow delta band (0.1–1 Hz) EEG activity before movement onset, which according to previous studies (Lew et al., 2012; Bulea et al., 2014), is unlikely to be contaminated by motion or muscular artifacts. Also, it has been found that ocular artifacts mainly affect the frontal EEG channels (Lew et al., 2012), which we did not use for detecting intent. Moreover, we used only the central EEG electrodes over the sensorimotor cortex, which are less likely to be corrupted by any muscular or ocular artifacts, if any. In addition, during offline calibration, we visually rejected noisy epochs from the training set to minimize their effects. Therefore, it is likely the effect of artifacts, if any, on the classifier's performance was negligible. It is also important to note that the manual selection of channels for analyzing MRCP used in this study will not scale for a larger number of patients. Therefore, in future work we plan to further investigate methods to automate channel selection personalized to each patient.

As seen in Figure 5E, the higher variations in the cross-validation accuracy of adaptive approach could have resulted from overfitting, since it uses smaller number of training samples (117 ± 16) per subject, which were retained using our criteria (see Section 2.3.1); whereas for the fixed window approach we used all training samples (154 ± 10) per subject. To overcome this limitation, larger number of training samples will be recorded and techniques for preventing overfitting of cross-validation data (Ng, 1997), will be considered in future. The proposed BMI performed well for less affected patients that could, in addition, benefit from EMG-gating. However, for more severely affected patients, other solutions than the one presented here may be required and should be explored further. Closed-loop control of BMI systems also has the potential to actively engage learning and adaptation and therefore change cortical activity (Orsborn and Carmena, 2013). The present feasibility study did not investigate this possibility. This question would be better addressed in a longitudinal study with a larger cohort of stroke patients.

## 5. Conclusions

This study demonstrates the feasibility of using movement related cortical potentials (MRCPs) recorded via EEG, to design a closed-loop BMI system for detecting motor intent of chronic stroke patients over multiple days and without recalibrating the BMI. Using the adaptive window approach proposed here together with calibration data from multiple days, we demonstrated closed-loop BMI performance, in spite of inter-trial variability and poor SNR of MRCPs. Our methods were validated in four stroke patients with varying severity of motor impairments, who were able to use the EEG-based BMI in real-time to control an upper-limb exoskeleton (MAHI-Exo II). We are currently testing our BMI approach in a clinical trial involving a larger population of chronic stroke patients to assess the potential benefits of using a personalized closed-loop BMI system for robot-based upper-limb rehabilitation.

## Author contributions

JC, MO, and GF conceived the project. NB, AV, EA, MO, GF, and JC designed the experiments. MO supplied the exoskeleton (MAHI Exo-II) and provided inputs to EA and JF for adapting the exoskeleton. AV, NY, and GF recruited the subjects. NB, AV, EA, and AB ran the experiments and collected the data. EA, AB were responsible for operating the exoskeleton. NY performed clinical assessment. CK, RG were responsible for MRI scans and interpretation. NB, BA, and EA analyzed the data with supervision by JC. NB performed the BMI design and optimization with input from JC. NB drafted and wrote the manuscript. BA performed the source analysis (refer Supplementary Materials). AV, AB, RG, MO, GF, and JC reviewed the drafts and made substantial comments. All authors read and approved the final manuscript.

### Conflict of interest statement

The authors declare that the research was conducted in the absence of any commercial or financial relationships that could be construed as a potential conflict of interest.
