# Supplementary material for: Design and Optimization of an EEG-Based Brain Machine Interface (BMI) to an Upper-Limb Exoskeleton for Stroke Survivors
Source: Front Neurosci. 2016 Mar 31;10:122. doi: 10.3389/fnins.2016.00122 (PMC4815250; doi:10.3389/fnins.2016.00122)
Supplement: Supplementary file 1 [file DataSheet1.pdf]

I. Supplementary Methods: Source Analysis

Cortical sources of movement related cortical potentials (MRCPs) were estimated for subjects S1, S2, and S4 (S3 declined MRI due to claustrophobia), on a trial by trial basis for days 4 and 5. The average source activation for each block and the grand-average across blocks for each day was then computed. The procedure for performing the source analysis is described below.

First, volumetric segmentation of the anatomical MR images was performed with BrainSuite software [A1] into the five different tissue segments within the head that consisted of scalp, outer skull, inner skull, cerebrospinal fluid (CSF) and white/gray matters. The anatomical regions of interest were further registered to a labeled atlas within the same software. Brainstorm, an open-source electromagnetic source imaging suite [A2], was then used for processing the anatomical and functional data for distributed source imaging. Raw EEG data from days 4 and 5 were pre-processed using the same approach as described in Section II-C1. EEG data analysis was primarily performed within Brainstorm and in addition the ICA toolbox from Curry 7 software (Compumedics, Charlotte, USA) was used to remove artifacts related to eye blinks, heart beats and bad blocks.

Further, co-registration between EEG and MRI data was done by projecting standard three dimensional EEG sensor positions on to the subject specific head surface and by applying operations such as geometrical translation, rotation and scaling. This procedure was very carefully designed in reference to video clips and images of the electrode positions of each subject that were obtained from each experiment and after visually matching visible electrodes (e.g. frontopolar, temporal and if visible the central electrodes) in MRI space.

Next, a three layer realistic head volume conductor model was prepared with OPENMEEG software [A3] by assigning constant isotropic electrical conductivity values representing the brain-skull interface, skull-skin interface and skin-air interface, where each layer is characterized by tessellations (vertices and triangles). For each layer a specific electrical conductivity value was defined as 0.33 S/m for the brain, 0.022 S/m for the skull and 0.33 S/m for the skin, respectively [A4], [A5].

Finally, cortically constrained source estimation was performed using the depth-weighted minimum norm estimate (MNE) [A6]. A source model containing over a set of ∼15249 elementary current dipoles was computed with constrained normal orientation to a cortex and distributed over the individual cortical surface. The source estimation maps were interpolated to the subject specific head surface using Brainstorm’s multilinear registration technique using default parameters. Statistically significant cortical maps of estimated sources were determined via Student's t-test (Bonferroni corrected). This was done by comparing individual sources of each trial with sources obtained from a pre-defined baseline. The baseline interval was taken from [-0.3s 0s] with respect to motor intent detection by the BMI.

Figures S-1, S-2, and S-3 show topographic distributions of activation loci for subjects S1, S2, and S4, respectively. Additionally, Tables S-1 and S-2 summarize the brain regions activated during user-driven (UD) and user-triggered (UT) modes for each subject. The variability in source activations across subjects is not surprising, given that the lesion size and location considerably varied across subjects. However, the results are only exploratory and a longitudinal study involving a larger patient population is being conducted to further understand the cortical sources of MRCPs in chronic stroke patients.

Supplementary references

[A1] D. W. Shattuck and R. M. Leahy, "BrainSuite: an automated cortical surface identification tool," *Med. Image Anal.*, vol. 6, no. 2, pp. 129–142, 2002.

[A2] F. Tadel, S. Baillet, J. C. Mosher, D. Pantazis, and R. M. Leahy, "Brainstorm: a user-friendly application for MEG/EEG analysis," *Comput. Intell. Neurosci.*, vol. 2011, p. 8, 2011.

[A3] A. Gramfort, T. Papadopoulo, E. Olivi, M. Clerc, and others, "OpenMEEG: opensource software for quasistatic bioelectromagnetics," *Biomed. Eng. Online*, vol. 9, no. 1, p. 45, 2010.

[A4] T. F. Oostendorp, J. Delbeke, and D. F. Stegeman, "The conductivity of the human skull: results of in vivo and in vitro measurements," *Biomed. Eng. IEEE Trans.*, vol. 47, no. 11, pp. 1487–1492, 2000.

[A5] Y. Zhang, W. van Drongelen, and B. He, "Estimation of in vivo brain-to-skull conductivity ratio in humans," *Appl. Phys. Lett.*, vol. 89, no. 22, p. 223903, 2006.

[A6] S. Baillet, J. C. Mosher, and R. M. Leahy, "Electromagnetic brain mapping," *Signal Process. Mag. IEEE*, vol. 18, no. 6, pp. 14–30, 2001.

 2

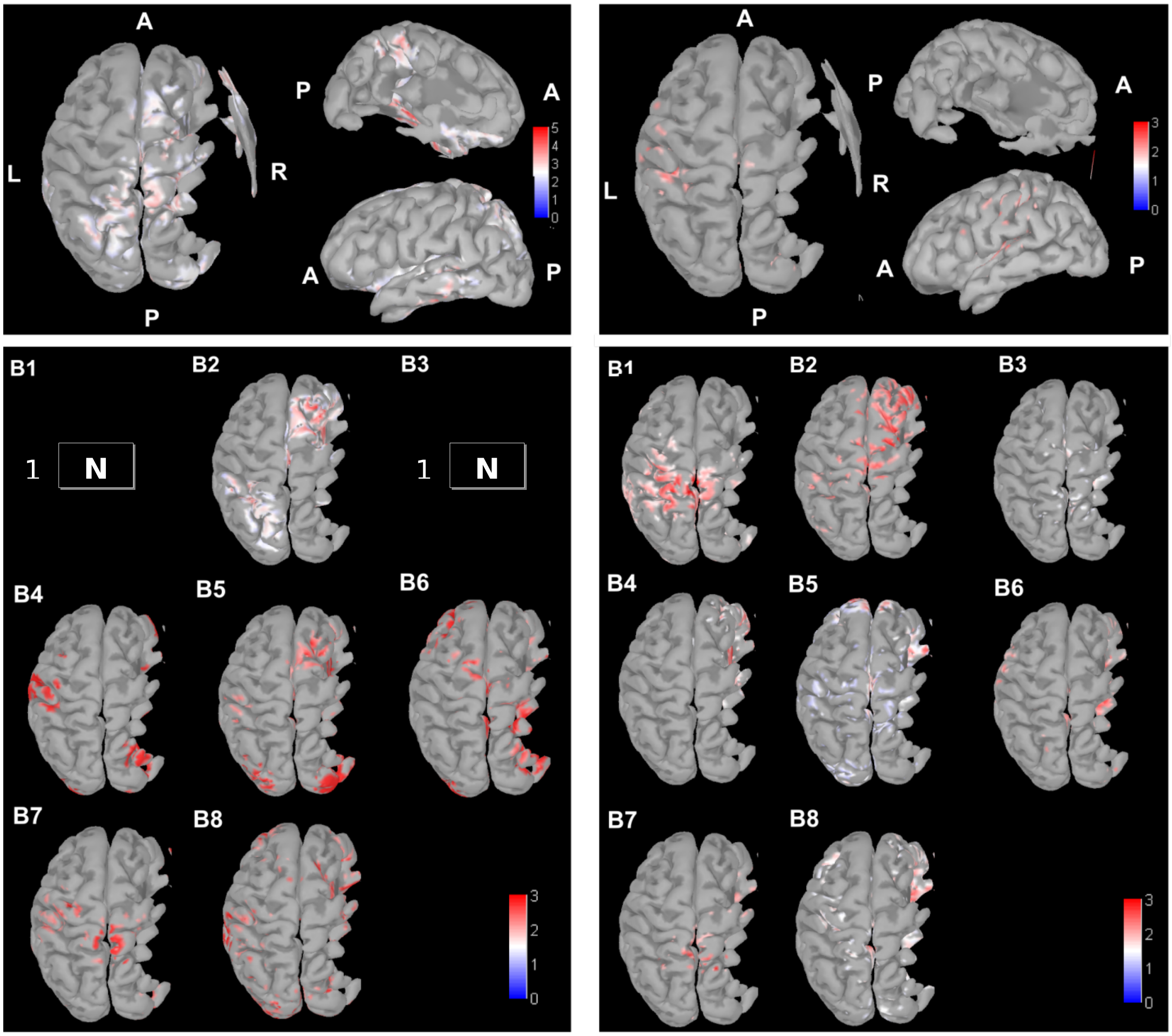

Figure S-1: Cortical source activity during days 4 (left column) and 5 (right column) for subject S1 (Left hand paretic), UT mode.

Left column:

Top panel: Grand averaged source activity across all blocks from Day 4. The activation patterns were localized on right precentral gyrus, primary motor cortex (BA 4) and left superior parietal cortex, posterior to somatosensory cortex (BA 7), primary auditory cortex, temporal lobe in the bank of the lateral sulcus (BA 42). Blocks for which source localization could not be performed because of errors in the recorded EEG data, are marked by NA.

Bottom panel: Block wise averaged source activity.

Right column:

Top panel: Grand averaged source activity across all blocks from Day 5. The activation patterns were localized on frontal lobe, postcentral gyrus (BA 6), right and left precentral gyrus, primary motor cortex (BA 4), and superior parietal cortex, posterior to somatosensory cortex (BA 7).

Bottom panel: Block wise averaged source activity.

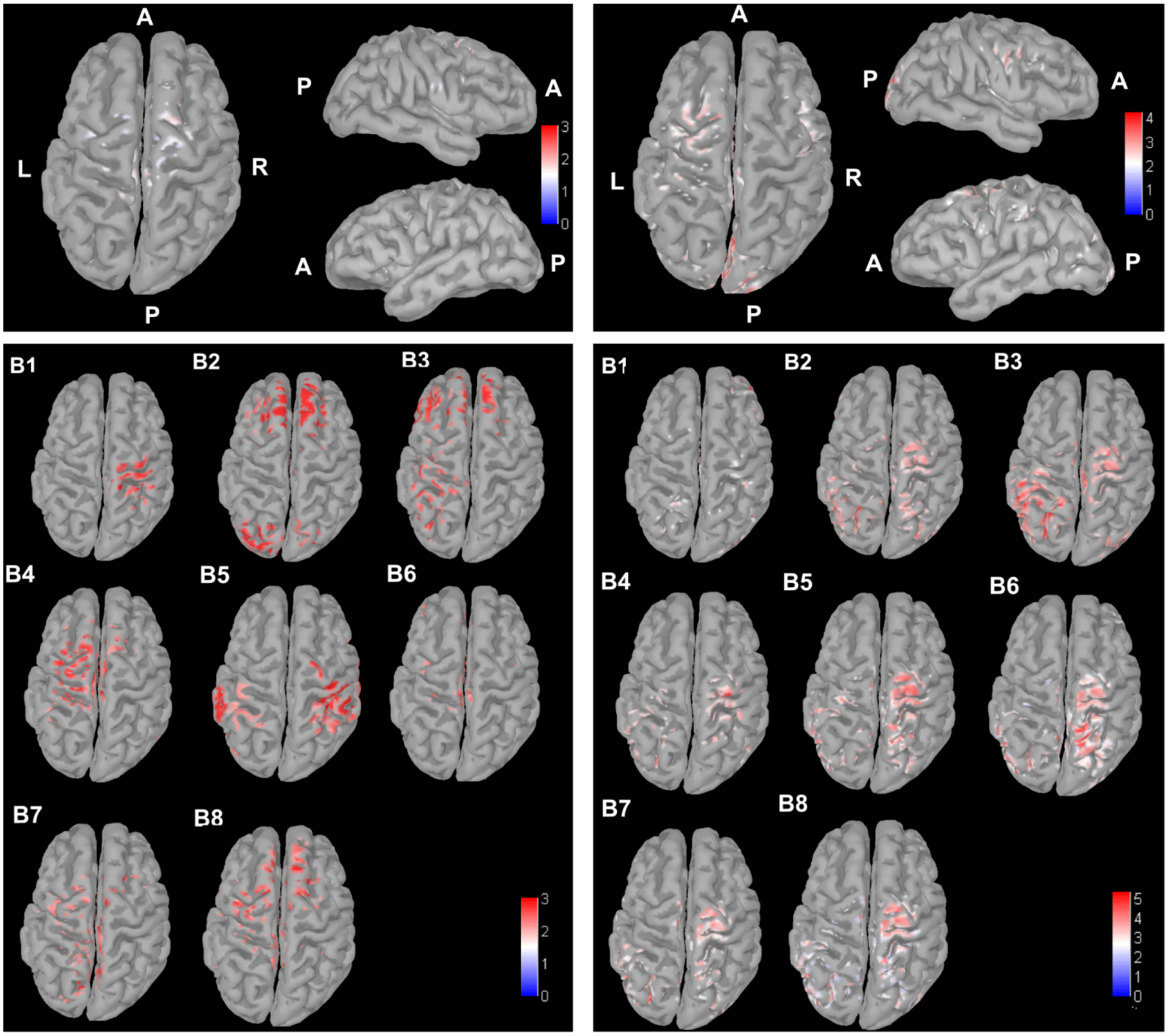

Figure S-2: Cortical source activity during day 4, UD mode (left column) and day 5, UT mode (right column) for subject S2 (Right hand paretic).

Left column:

Top panel: Grand averaged source activity across all blocks from Day 4. The activation patterns were localized on right superior frontal gyrus, prefrontal cortex (BA 8), right precentral gyrus, primary motor cortex (BA 4), right/left postcentral gyrus, primary somatosensory cortex (BA 5).

Bottom panel: Block wise averaged source activity.

Right column:

Top panel: Grand averaged source activity across all blocks from Day 5. The activation patterns were localized on right superior frontal gyrus, prefrontal cortex (BA 8), right precentral gyrus, primary motor cortex (BA 4) and right/left postcentral gyrus, primary somatosensory cortex (BA 5).

Bottom panel: Block wise averaged source activity.

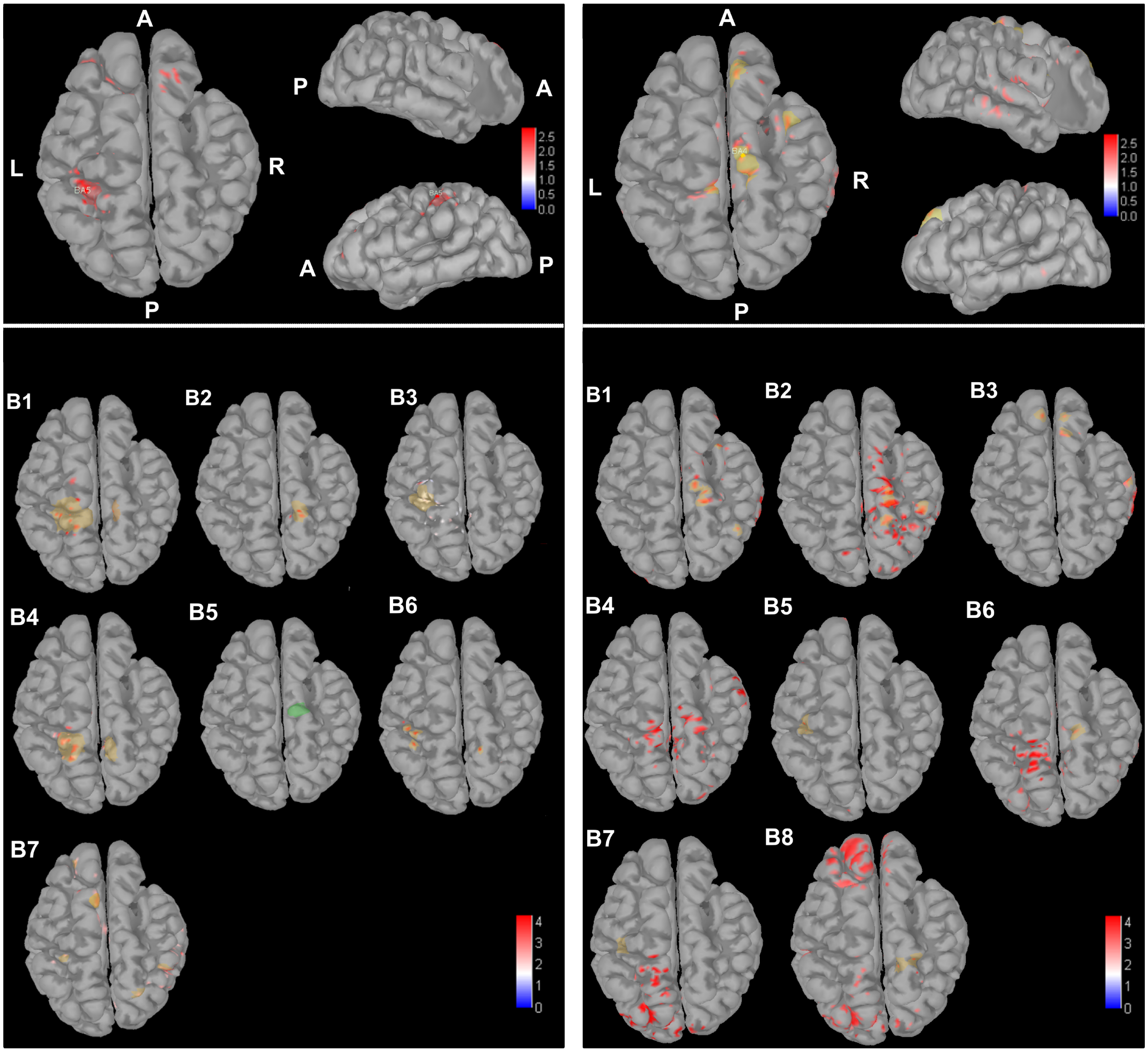

Figure S-3: Cortical source activity during day 4, UD mode (left column) and day 5, UT mode (right column) for subject S4 (Left hand paretic).

Left column:

Top panel: Grand averaged source activity across all blocks from Day 4. The activation patterns were localized on primary somatosensory cortex (BA 5) and other sources were localized on right superior frontal area (BA 9).

Bottom panel: Block wise averaged source activity. Sources on Blocks B2, B5 show significant activation on right primary motor cortex (BA 4) and B1, B4, B6 show involvement of bilateral cortical areas of primary sensorimotor (BA 4, BA 5). The source activations for B3 were localized on left primary motor cortex, and for B7 activation patterns were not focal.

Right column:

5

Top panel: Grand averaged source activity across all blocks from Day 5. The activation patterns were localized on right primary motor cortex (BA 4) and on right superior frontal area (BA 9) as well as on right temporal lobe (BA 20, 21, 22).
Bottom panel: Sources across Blocks B1, B2, B4, B6, B8 show significant activation both on sensory and motor cortices (BA 2, 4 ,5) and B5, B7 show involvement of cortical areas responsible for integration of visual and motor information (BA 5, 7).

Table S-1: Summary of the brain regions activated during the user-driven mode when motor intent was detected by closed-loop BMI controller. Grand averaged activity across all blocks is listed.

Subjects	Cerebral Regions and Brodmann Areas	Functional Area Descriptions	t-statistic ($p < 0.05$)
S2	Right superior frontal gyrus, prefrontal cortex (BA 8)<br><br>Right precentral gyrus, primary motor cortex (BA 4);<br><br>Right/left postcentral gyrus, primary somatosensory cortex (BA 5);	BA 8: It forms the premotor cortex together with parts of BA 6, Planning of movement.<br><br>BA 4 : Control of voluntary movements; Contains motor homunculus;<br><br>BA 5: Somatosensory processing and association, muscle imagery	4.671
S4	Left postcentral gyrus, primary somatosensory cortex (BA 5);<br><br>Right and left superior frontal gyrus, prefrontal cortex (BA 9)	BA 5: Somatosensory processing and association, muscle imagery<br><br>BA 9: Executive function, cognitive control	2.837

Abbreviation: BA - Brodmann area

6

Table S-2: Summary of the brain regions activated during the user-triggered mode when motor intent was detected by closed-loop BMI controller. Grand averaged activity across all blocks is listed.

N	Cerebral Regions and Brodmann Areas	Functional Area Descriptions	t-statistic ($p < 0.05$)
S1 (Day 4)	Right precentral gyrus, primary motor cortex (BA 4);<br><br>Left superior parietal cortex, posterior to somatosensory cortex (BA 7),<br><br>Primary auditory cortex, Temporal lobe in the bank of the lateral sulcus (BA 42)	BA 4 : Control of voluntary movements; Contains motor homunculus;<br><br>BA 7: Integration of visual and motor information; Visuo-motor coordination<br><br>BA 42: Early processing auditory information	3.186
S1 (Day 5)	Frontal lobe, Postcentral gyrus (BA 6)<br><br>Right and left precentral gyrus, Primary motor cortex (BA 4)<br><br>Superior parietal cortex, Posterior to Somatosensory cortex (BA 7)	BA 6: Sensory guidance of movement<br>Control of proximal and trunk muscles of the body<br><br>BA 4 : Control of voluntary movements; Contains motor homunculus;<br><br>BA 7: Integration of visual and motor information. Together with BA5, it forms the secondary somatosensory cortex. Visuo-motor coordination.	7.225
S2	Right superior frontal gyrus, prefrontal cortex (BA 8)<br><br>Right precentral gyrus, primary motor cortex (BA 4);<br><br>Right/left postcentral gyrus, primary somatosensory cortex (BA 5);	BA 8 : It forms the premotor cortex together with parts of BA 6, Planning of movement.<br><br>BA 4 : Control of voluntary movements; Contains motor homunculus;<br><br>BA 5: Somatosensory processing and association, muscle imagery	4.671

7

S4	Frontal lobe, Postcentral gyrus (BA 6)<br><br>Right and left precentral gyrus, Primary motor cortex (BA 4)<br><br>Superior parietal cortex, Posterior to Somatosensory cortex (BA 7)	BA 6: Sensory guidance of movement<br>Control of proximal and trunk muscles of the body<br><br>BA 4 : Control of voluntary movements; Contains motor homunculus;<br><br>BA 7: Integration of visual and motor information. Together with BA5, it forms the secondary somatosensory cortex. Visuo-motor coordination.	7.225

8

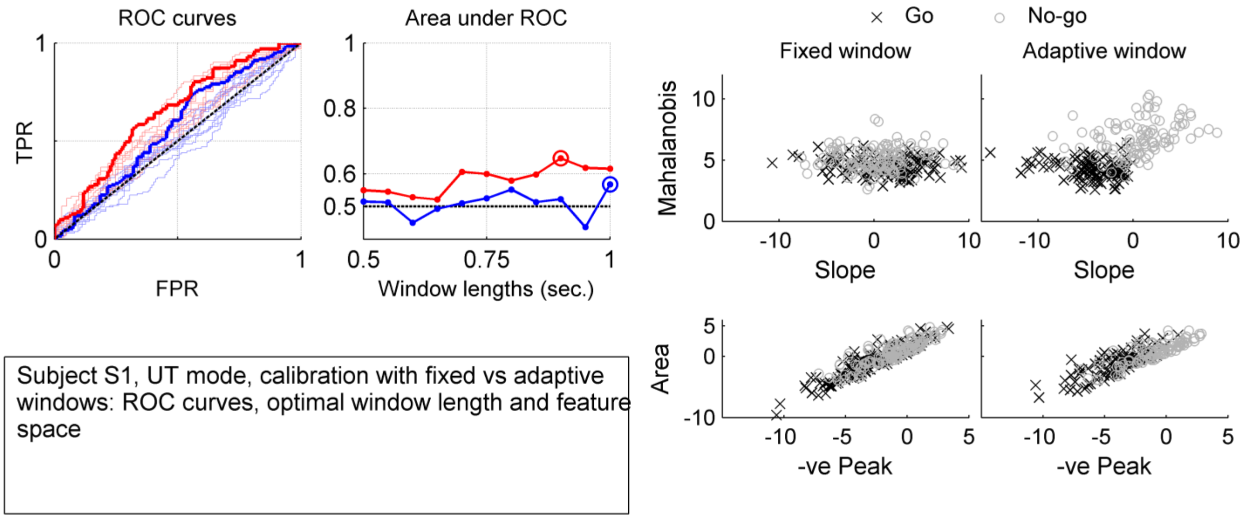

Figure S-4: Comparison of fixed and adaptive window techniques for optimal window selection for subject S1, user-triggered (UT) mode

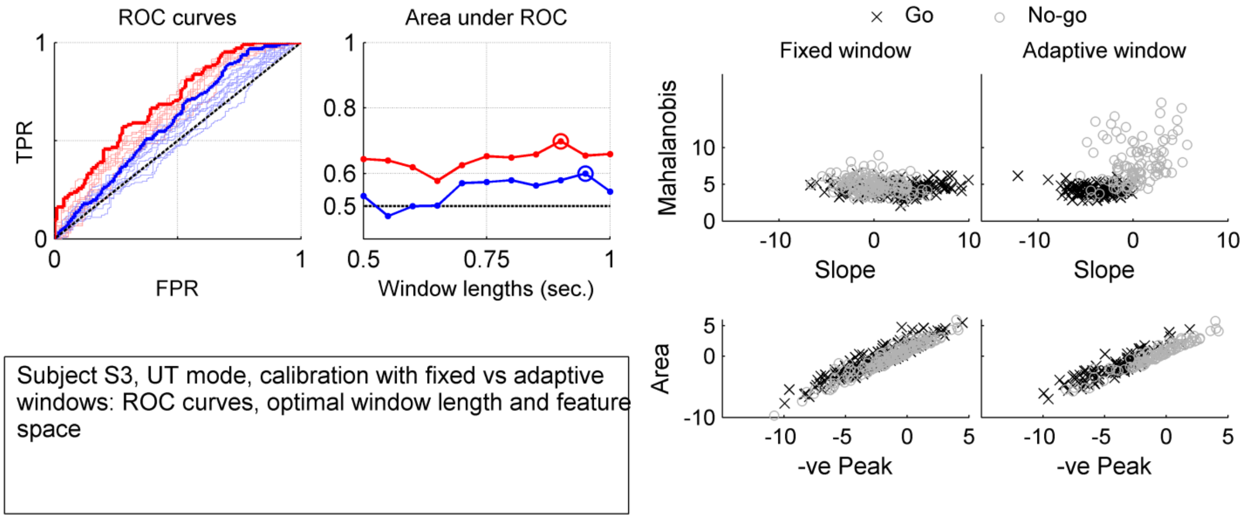

Figure S-5: Comparison of fixed and adaptive window techniques for optimal window selection for subject S3, user-triggered (UT) mode

9

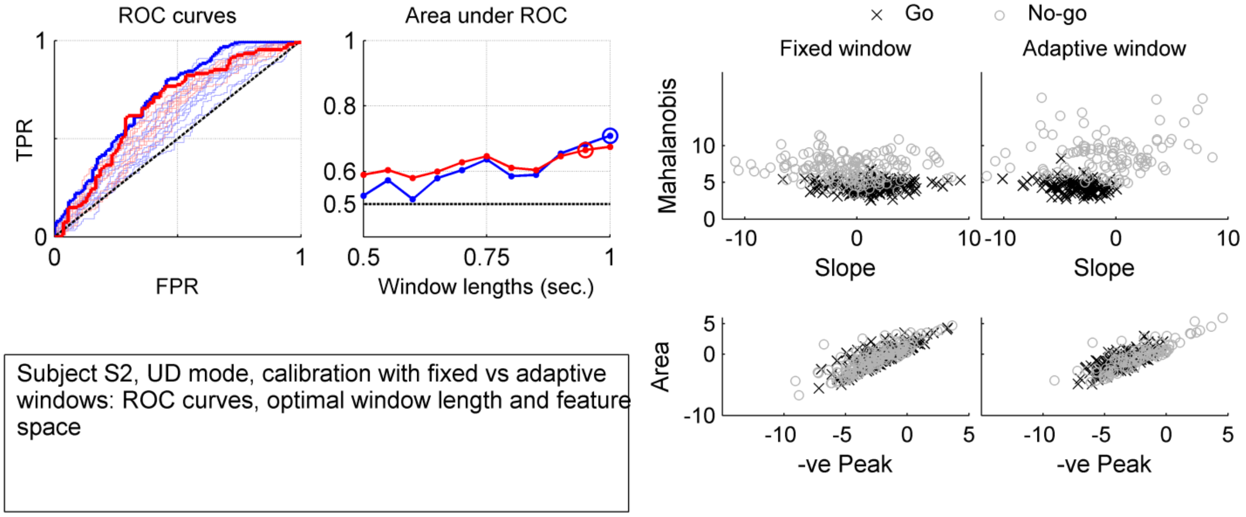

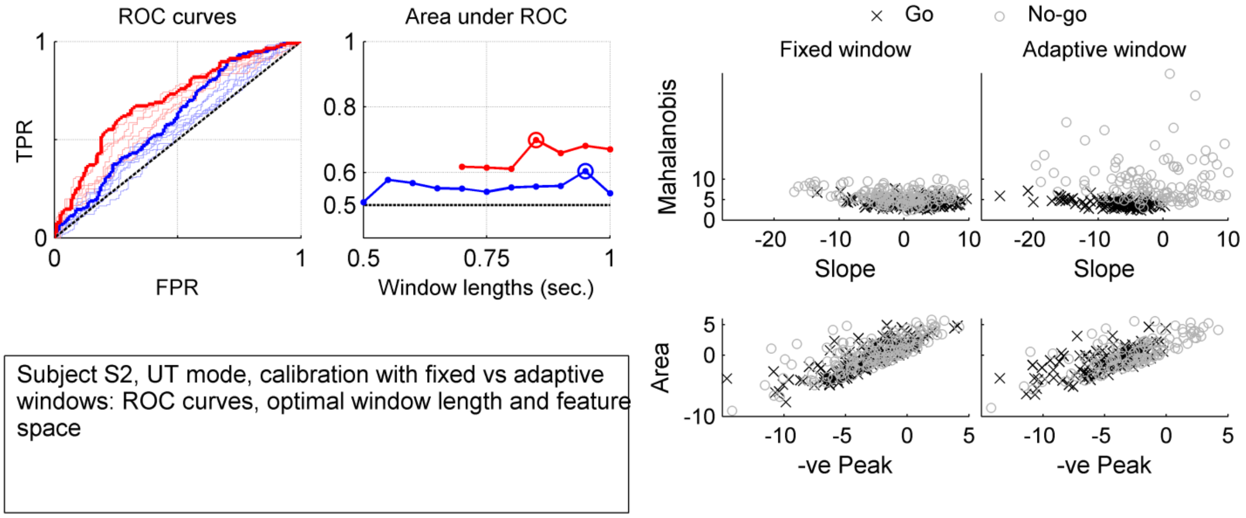

Figure S-6: Comparison of fixed and adaptive window techniques for optimal window selection for subject S2, user-driven (UD) (top) and user-triggered (UT) (bottom) modes

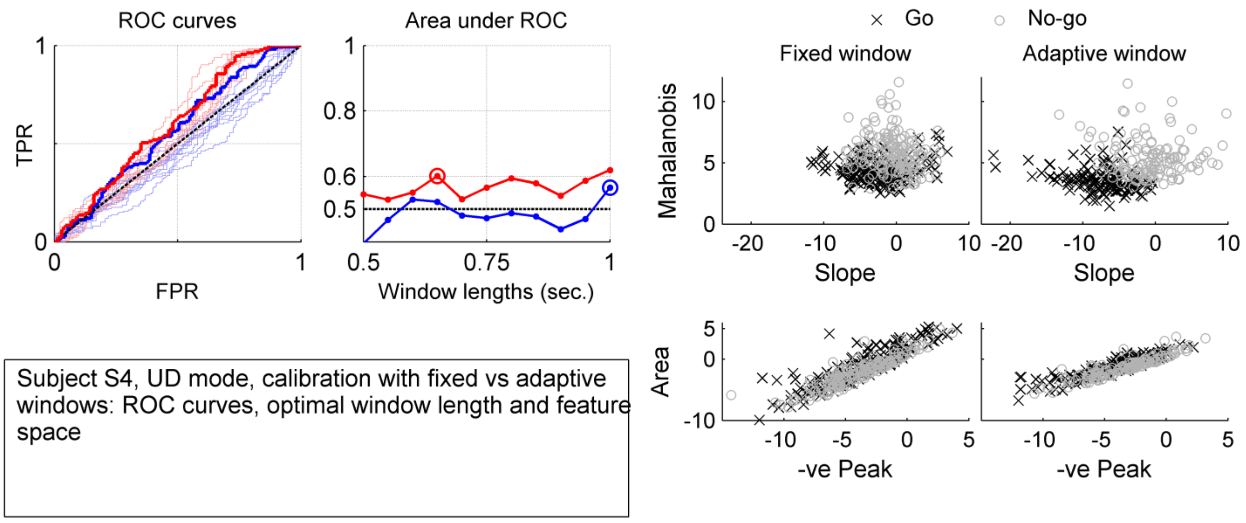

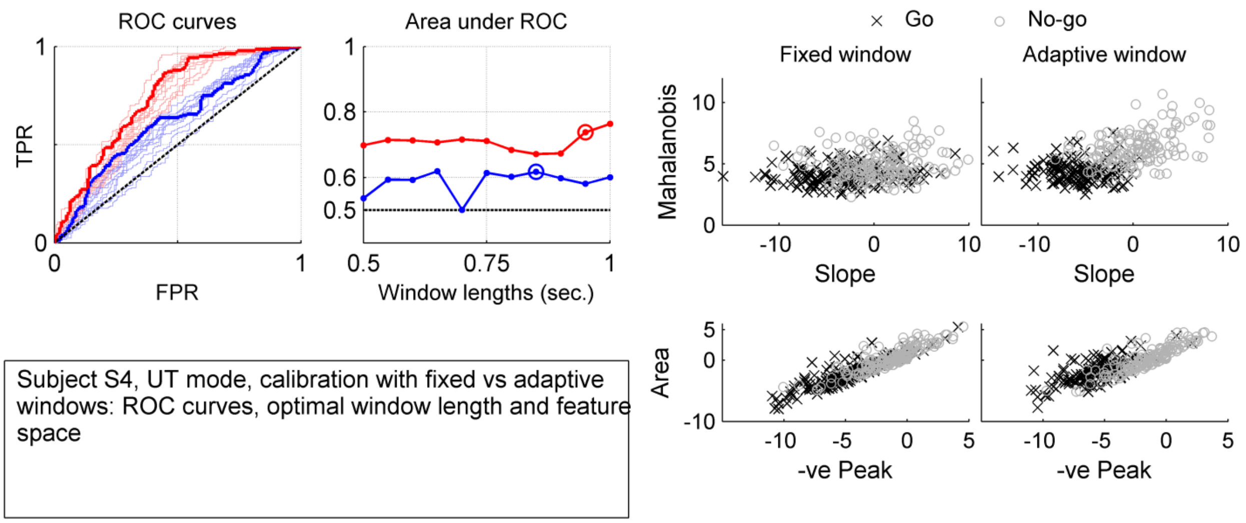

Figure S-7: Comparison of fixed and adaptive window techniques for optimal window selection for subject S4, user-driven (UD) (top) and user-triggered (UT) (bottom) modes. Results for subject S4, UT mode are also reported in the paper.

11

TABLE S-3
MEAN (SD) VALUES FOR METRICS USED TO EVALUATE BMI PERFORMANCE DURING CLOSED-LOOP TESTING

Subject	True Positive Rate (%)		False Positive Rate (%)		Detection latency (s)		Subject ratings[#]	
	Day 4	Day 5	Day 4	Day 5	Day 4	Day 5	Day 4	Day 5
S1[†]	59 (28)	60 (9)	65 (32)	59 (39)	-0.50 (0.43)	-0.46 (0.40)	2.29 (1.46)	2.48 (1.47)
S2[‡]	81 (17)	60 (17)	0 (0)	13 (25)	-0.68 (0.78)	-0.48 (0.56)	4.09 (1.36)	3.17 (1.72)
S3[†]	52 (16)	73 (10)	35 (44)	28 (30)	-0.35 (0.39)	-0.61 (0.32)	2.93 (1.78)	3.27 (1.56)
S4[‡]	59 (9)	79 (12)	8 (14)	6 (18)	-0.31 (0.50)	0.30 (0.90)	3.29 (1.77)	3.83 (1.42)
Overall mean (SD) per day	62.71 (21.43)	67.08 (14.55)	27.74 (37.46)	27.50 (35.64)	-0.48 (0.18)	-0.26 (0.43)	3.15 (1.73)	3.15 (1.63)
Overall mean (SD) across days	64.86 (18.35)		27.62 (36.37)		-0.367 (0.328)		3.15 (1.68)	

[#] 5-point subject rating, ranging from 1-5, where 1 implied completely inaccurate and 5 implied completely accurate BMI decision for a trial.
[†] BMI calibrated using user-triggered mode only was used for closed-loop testing on both days.
[‡] BMI calibrated using user-driven and user-triggered modes were used for closed-loop testing on days 4 and 5, respectively.
